# New Terpenoids and Polyphenolic Profile of *Carpesium cernuum* L. of European Origin

**DOI:** 10.3390/molecules30122506

**Published:** 2025-06-07

**Authors:** Janusz Malarz, Danuta Jantas, Klaudia Jakubowska, Ryszard Bugno, Anna K. Kiss, Anna Stojakowska

**Affiliations:** 1 Maj Institute of Pharmacology, Polish Academy of Sciences, Smętna Street 12, 31-343 Kraków, Poland; malarzj@if-pan.krakow.pl (J.M.); jantas@if-pan.krakow.pl (D.J.); bugno@if-pan.krakow.pl (R.B.); 2 Department of Pharmaceutical Biology, Medical University of Warsaw, Banacha Street 1, 02-097 Warsaw, Poland; akiss@wum.edu.pl

**Keywords:** caffeic acid derivatives, *Carpesium cernuum* L., cytotoxicity, germacranolides, hydroxycinnamates, neuroblastoma, neuroprotection, thymol derivatives

## Abstract

*Carpesium cernuum* L., the most widespread representative of the genus *Carpesium*, has been traditionally used in some regions of Asia as a remedy for various ailments or as a vegetable. Although the plant is distributed in Europe, there is no data on its medicinal use in this part of the world. The chemical composition of European *Carpesium cernuum* L. has remained unknown until now, except for the compositions of essential oils distilled from the roots and aerial parts of the plant. Polyphenolic profiles of hydroalcoholic extracts from *C. cernuum* were studied using the HPLC-MS^n^ technique. The analysis revealed the presence of 24 hydroxycinnamates, which were dominated by caffeoylquinic and caffeoylhexaric acids. Moreover, fractionation of the chloroform extracts from the plant led to the isolation of three new compounds, 8α-angeloyloxy-4β-hydroxy-5β-(3-methylbutyryloxy)-9-oxo-germacran-6α,12-olide, 9β-angeloyloxy-4β,8α-dihydroxy-5β-(3-methylbutyryloxy)-3-oxo-germacran-6α,12-olide, and a dihydrobenzofuran derivative, together with twelve known compounds. 8-Hydroxy-9,10-diisobutyryloxythymol, a monoterpenoid thymol derivative from the roots of the plant, was evaluated for potential neuroprotective and cytotoxic activities using differentiated and undifferentiated SH-SY5Y neuroblastoma cells. At a concentration range of 1–10 μM, the compound provided partial (up to 50%) protection against H_2_O_2_-induced cell damage in the undifferentiated cells. At concentrations higher than 25 μM, the monoterpenoid significantly reduced the viability of the cells (IC_50_: 65.7 μM for the undifferentiated cells and 40.9 μM for the differentiated cells).

## 1. Introduction

Plants of the genus *Carpesium* (Asteraceae; subtribe: Inuleae-Inulinae) are mostly native to China, Japan, and Korea and some of them are endemic to China. As a member of the Inuleae-Inulinae subtribe, the genus is a close relative to medicinal plants such as *Blumea balsamifera* (L.) DC., *Dittrichia viscosa* (L.) Greuter, *Inula japonica* Thunb., *I*. *helenium* L., *Pulicaria undulata* (L.) C.A.Mey., and others. *Carpesium cernuum* L. is the most widespread species of the *Carpesium* genus; it is native to Eurasia and is currently distributed in Europe, Asia, Australia, and Oceania. The plant is a biennial or perennial herb that inhabits wasteland and mountain slopes below 3000 m.a.s.l. It can grow up to 50–80 cm tall, and has an erect branched stem and solitary capitula (15–18 mm), which are subtended by many linear-lanceolate leaves [1,2,3]. In China and Kashmir, extracts and decoctions from the whole plant are utilized as medicines, mainly to relieve inflammation and pain. Local communities in China and India use the whole herb or its aerial parts to prepare meals and beverages [4,5,6,7,8,9,10]. Recently, anti-inflammatory and tumor migration inhibitory effects of extracts from *C. cernuum* have been described [11,12].

Majority of the sesquiterpenoid metabolites from *C. cernuum* demonstrated cytotoxic activity towards different human cancer cell lines in vitro [4]. However, their selectivity and the mechanisms of action remain less explored. Germacranolides from the active fractions of the ethanol extract from *C. cernuum* reduced both the migration and invasion of breast cancer cells (MDA-MB-231) [11] and may be partly responsible for the antimetastatic potential of the whole extract. Incaspitolide A, a germacrane-type sesquiterpene lactone isolated from *C. cernuum*, induced apoptosis in BPH-1 (benign prostatic hyperplasia) cells and PC-3 (prostate cancer) cells via the PI3K/Akt (phosphatidylinositol 3-kinase/protein kinase B) pathway [13,14]. Carpescernolides C–E demonstrated significant activity against three different strains of human leukemia cells (HEL, KG-1a, and K562) by inducing apoptosis via the activation of extracellular signal-activated kinase (ERK) [15]. Another *C. cernuum* germacranolide, cernuumolide J, inhibited the proliferation of HEL cells, inducing cell cycle arrest at the G2/M phase and subsequent apoptosis through the Janus kinase 2 signal transducer/activator of transcription 3 (JAK2/STAT3) and mitogen-activated protein kinase (MAPK) pathways [16]. Carabrone, a sesquiterpene lactone of a different structural type, inhibited the proliferation and migration of pancreatic cancer cells (SW1990) through the up regulation of a protein kinase from the casein kinase I protein family (CSNK1E) and activation of the Hippo signaling pathway, ultimately leading to ferroptosis [17].

Although many studies on the isolation of terpenoids, and more scarcely polyphenolics, from *C. cernuum* collected in China and Korea have been published [4,15,18,19,20,21], data on the secondary metabolites produced by the plants of European origin, except for the components of their essential oils [22], are not available. The polyphenolic composition of *C. cernuum* extracts has never been studied in detail using advanced analytical techniques.

Assessment of the potential value of *C. cernuum* grown in Europe as a source of biologically active constituents that could be used in therapeutics and the prevention of human diseases was the objective of the current study.

## 2. Results

### 2.1. Caffeic Acid Derivatives in Roots and Aerial Parts of C. cernuum

**Figure 1:**
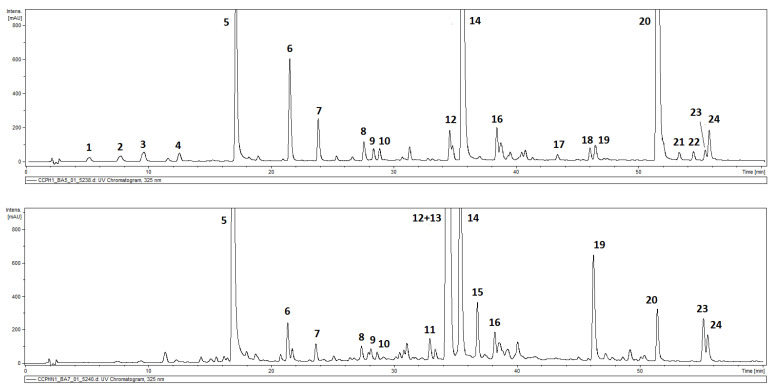
HPLC-UV chromatogram of *Carpesium cernuum* L. extracts (2 μL of 10 mg/mL extract injected) acquired at 325 nm: upper part—roots; lower part—aerial parts. Compounds: **1**—caffeoylhexaric acid (I); **2**—caffeoylhexaric acid (II); **3**—caffeoylhexaric acid (III); **4**—caffeoylhexaric acid (IV); **5**—5-*O*-caffeoylquinic acid; **6**—dicaffeoylhexaric acid (I); **7**—dicaffeoylhexaric acid (II); **8**—dicaffeoylhexaric acid (III); **9**—unidentified phenolic acid derivative; **10**—dicaffeoylhexaric acid (IV); **11**—3;4-di-*O*-caffeoylquinic acid; **12**—1;5-di-*O*-caffeoylquinic acid; **13**—3;5-di-*O*-caffeoylquinic acid; **14**—tricaffeoylhexaric acid (I); **15**—4;5-di-*O*-caffeoylquinic acid; **16**—tricaffeoylhexaric acid (II); **17**—isobutyryl-dicaffeoylhexaric acid; **18**—isobutyryl-tetracaffeoylhexaric acid; **19**—tetracaffeoylhexaric acid; **20**—isobutyryl-tricaffeoylhexaric acid (I); **21**—isobutyryl-tricaffeoylhexaric acid (II); **22**—isobutyryl-tricaffeoylhexaric acid (III); **23**—2-methylbutyryl/isovaleryl-tricaffeoylhexaric acid (I); **24**—2-methylbutyryl/isovaleryl-tricaffeoylhexaric acid (II).

Hydroalcoholic extracts (70% MeOH) from the underground parts and leaves of *C. cernuum* were analyzed for their polyphenolic components using the HPLC-DAD-MS^n^ technique. Twenty-three compounds in total were tentatively identified in the extracts and all of them demonstrated absorption maxima at 323–328 nm (caffeic acid derivatives). Twenty of the detected hydroxycinnamates were found in the roots of the plant and fifteen were found in the aerial parts (Figure 1, Table 1). Compounds 1–4 (*m*/*z* = 371 [M−H]^−^), detected exclusively in the roots, showed the cleavage of one caffeoyl moiety [M−H-162]^−^, resulting in an *m*/*z* 209 fragment and were identified as isomers of caffeoylhexaric acid. Peak 5 (*m*/*z* = 353 [M−H]^−^) was identified as a signal of 5-*O*-caffeoylquinic acid (5-CQA in IUPAC numbering system), whereas compounds 11–13 and 15 (*m*/*z* = 515 [M−H]^−^), based on their fragmentation patterns (Table 1), were recognized as four di-*O*-caffeoyl quinic acids (DCQAs), namely, 3,4-; 1,5-, 3,5- and 4,5-di-*O*-caffeoylquinic acids [23,24,25]. Peaks 6–8, 10, 14, 16, and 19, representing compounds which showed a cleavage of two, three, or four caffeoyl [M−H-(2–4 × 162)]^−^ moieties resulting in an *m*/*z* 209 fragment, were assigned to hexaric acid derivatives. Compounds 6–8 and 10 (*m*/*z* = 533 [M−H]^−^) were identified as di-*O*-caffeoylhexaric acid I (peak 6), di-*O*-caffeoylhexaric acid II (peak 7), di-*O*-caffeoylhexaric acid III (peak 8), and di-*O*-caffeoylhexaric acid IV (peak 10). Peaks 14 and 16 (*m*/*z* = 695 [M−H]^−^) corresponded to tri-*O*-caffeoylhexaric acid I and tri-*O*-caffeoylheharic acid II, respectively, whilst peak 19 (*m*/*z* = 857 [M−H]^−^) represented tetra-*O*-caffeoylhexaric acid [25,26,27,28]. Compounds represented by peaks 17 and 20–22 were isobutyryl-dicaffeoylhexaric acid and isobutyryl-tricaffeoylhexaric acids I–III based on the *m*/*z* values of their quasimolecular ions (603 [M−H]^−^ and 765 [M−H]^−^, respectively) and fragmentation ions at *m*/*z* 441, 423, and 279 [25,26,27,28]. The compound corresponding to peak 18 (*m*/*z* = 927 [M−H]^−^), with a similar fragmentation pattern to those of 20–22, was identified as isobutyryl-tetracaffeoylhexaric acid. The fragmentation patterns of compounds 23 and 24 (*m*/*z* = 779 [M−H]^−^) were similar to those of 20–22, except for the fact that the masses of the quasimolecular ion and fragmentation ions at *m*/*z* 617, 455, and 293 were fourteen units higher than the corresponding ones from the isobutyryl-tricaffeoylhexaric acids. Therefore, compounds 23–24 were tentatively identified as 2-methylbutyryl- or 3-methylbutyryltricaffeoylhexaric acids. Moreover, an unidentified caffeoylglucose derivative was detected in both the roots and aerial parts of *C. cernuum* [25,26,27,28]. The leaves and roots of *C. cernuum* contained 91.3 ± 4.4 mg GA eq/g DW and 50.6 ± 4.0 mg GA eq/g DW of phenolics, respectively. The chlorogenic acid (5-CQA) content in the leaves of the plant was estimated to be 1.14 ± 0.07%.

### 2.2. Terpenoids from the Aerial Parts and Roots of C. cernuum

**Figure 2:**
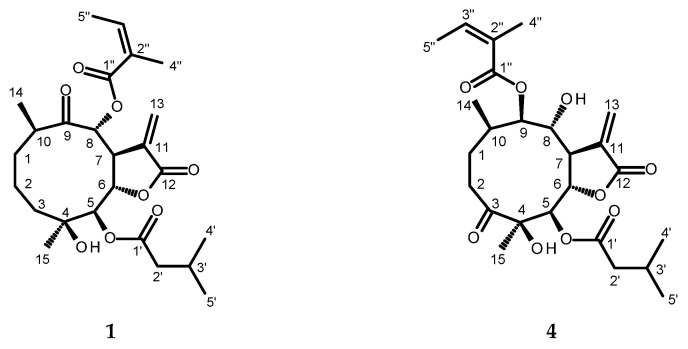
Chemical structures of 8*α*-angeloyloxy-4*β*-hydroxy-5*β*-(3-methylbutyryloxy)-9-oxo-germacran-6*α*,12-olide (**1**) and 9*β*-angeloyloxy-4*β*,8*α*-dihydroxy-5*β*-(3-methylbutyryloxy)-3-oxo-germacran-6*α*,12-olide (**4**) from aerial parts of *C. cernuum*.

After the chromatographic separation of the chloroform extract from the aerial parts of *C. cernuum*, two previously undescribed sesquiterpene lactones, 8*α*-angeloyloxy-4*β*-hydroxy-5*β*-(3-methylbutyryloxy)-9-oxo-germacran-6*α*,12-olide (**1**) and 9*β*-angeloyloxy-4*β*,8*α*-dihydroxy-5*β*-(3-methylbutyryloxy)-3-oxo-germacran-6*α*,12-olide (**4**), together with six known terpenoids (4*β*-hydroxy-5*β*-isobutyryloxy-8*α*-(3-methylbutyryloxy)-9-oxo-germacran-6*α*,12-olide (**2**) [29,30], 4*β*,8*α*-dihydroxy-5*β*-isobutyryloxy-9*β*-(3-methylbutyryloxy)-3-oxo-germacran-7*β*,12-olide (**3**) [31], divarolide E (**5**) [32], incaspitolide D (**7**) [29,31,33], 5*β*-angeloyloxy-4*β*,8*α*-dihydroxy-9*β*-isobutyryloxy-3-oxo-germacran-7*β*,12-olide (**8**) [34], and loliolide (**6**) [35]) were identified. The structures of the new compounds (**1** and **4**, see Figure 2) were deduced based on the analysis of their spectral data (HRESIMS, 1D- and 2D-NMR experiments, and specific rotation measurements).

**Figure 3:**
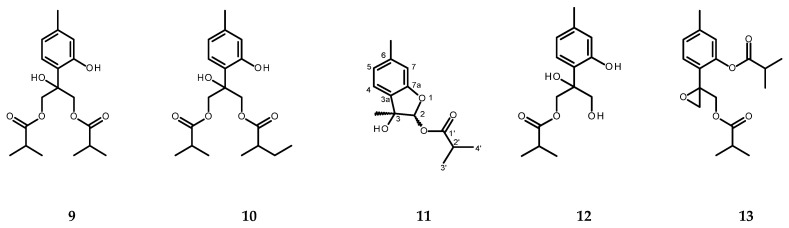
Chemical structures of thymol derivatives (**9**, **10**, **12**, and **13**) and carpesibenzofuran (**11**) from roots of *C. cernuum*.

The chloroform extract from the roots of *C. cernuum* after the chromatographic fractionation yielded a previously unknown structural analog of eupatobenzofuran [36], 3-hydroxy-3,6-dimethyl-2,3-dihydrobenzofuran-2-yl isobutyrate (carpesibenzofuran, **11**), as well as various known thymol derivatives: 8-hydroxy-9,10-diisobutyryloxythymol (**9**) [37], 8-hydroxy-9-isobutyryloxy-10-(2-methylbutyryloxy)-thymol (**10**) [38], 8,9-dihydroxy-10-isobutyryloxythymol (**12**) [39], and 8,9-epoxy-10-isobutyryloxythymol isobutyrate (**13**) [40] (see Figure 3 for structures). The latter compound was the dominant constituent of the freshly prepared chloroform extract from the roots of *C. cernuum* (0.096 ± 0.003% of the root dry weight) and could be easily detected in the ^1^H NMR spectrum of the extract (Supplementary Materials, Figure S1). Moreover, the root extract contained stigmasterol (**14**) and syringaldehyde (**15**).

#### Structure Elucidation

Compound **1** was isolated as a white amorphous solid. An adduct ion peak at *m*/*z* 487.2304 [M + Na]^+^ that was observed in the HRESIMS spectrum of **1** (positive ion mode; see Supplementary Materials, Figure S2) corresponded to the molecular formula of C_25_H_36_O_8_Na^+^ (calculated mass: 487.2308). The molecular formula of **1**, established as C_25_H_36_O_8_ (Figure 2), indicated eight degrees of unsaturation that could be accounted for by two ring systems, two olefinic double bonds, and four carbonyl groups. The ^1^H and ^13^C NMR spectra of 1 (Table 2; Supplementary Materials, Figures S3, S4, and S6) resembled those of 8*α*-angeloyloxy-4*β*-hydroxy-5*β*-isobutyryloxy-9-oxo-germacran-7*β*,12*α*-olide [30,31] except for the signals of the isobutyric substituent that were replaced by those of the 3-methylbutyric (isovaleric) group (Supplementary Materials, Table S1). The ^1^H-^1^H COSY spectrum (Supplementary Materials, Figure S5) demonstrated the presence of two partial structure sequences, CH_2_(3)-CH_2_(2)-CH_2_(1)-CH(10)-CH_3_(14) and CH(5)-CH(6)-CH(7)-CH(8), as well as 3-methylbutyryloxy CH_3_(4′,5′)-CH(3′)-CH_2_(2′) and angeloyloxy CH_3_(5”)-CH(3”) groups. The HMBC correlations of H-5 with δ_C_ 173.4 (ester carbonyl of 3-methylbutyryloxy group) and H-8 with δ_C_ 165.7 (ester carbonyl of angeloyloxy group) confirmed the deduced substitution pattern (Table 2; Supplementary Materials, Figure S7). The specific rotation measurement and the results of the NOESY experiment that demonstrated H-5/H_3–_15, H-7/H-5, H-7/H-10 and H-8/H6 correlations (Supplementary Materials, Figure S8) confirmed that the configuration of **1** corresponded to that established by Gao et al. [31] and Zhang et al. [30] for the series of analogous compounds (*Carpesium* germacranolides structural subtype III). Thus, the structure of 1 was determined to be 8*α*-angeloyloxy-4*β*-hydroxy-5*β*-(3-methylbutyryloxy)-9-oxo-germacran-6*α*,12-olide.

Compound **4** was obtained as colorless needles. A pseudomolecular ion peak at *m*/*z* 479.2282 [M−H]^−^ in the HRESIMS spectrum of **4** (negative ion mode; Supplementary Materials, Figure S9) indicated a molecular formula of C_25_H_35_O_9_ (calculated mass: 479.2281). The molecular formula of the compound (C_25_H_36_O_9_) corresponded to that of cardivarolide G [33] (*Carpesium* germacranolide structural subtype IV) and indicated eight degrees of unsaturation, just like compound **1**. In addition to the molecular formula, the ^1^H and ^13^C NMR spectra (Table 3; Supplementary Materials, Figures S10 and S11, Table S1) also suggested that **4** is an isomer of cardivarolide G. The most striking difference was observed when comparing the ^1^H NMR spectra of **4** and cardivarolide G. The chemical shift value for the proton at C-5 of **4** (δ_H_ 5.41) was significantly smaller than that of the corresponding proton in the cardivarolide G molecule (δ_H_ 5.52), whereas the signal of the proton at C-9 of **4** (δ_H_ 5.26) was shifted downfield in comparison with the corresponding signal in the cardivarolide G proton NMR spectrum (δ_H_ 5.18). This, together with the HMBC correlations of H-5 with δ_C_ 172.4 (ester carbonyl of 3-methylbutyryloxy group) and H-9 with δ_C_ 167.8 (ester carbonyl of angeloyloxy group) suggested that **4** is a positional isomer of cardivarolide G (see Figure 2), namely, 9*β*-angeloyloxy-4*β*,8*α*-dihydroxy-5*β*-(3-methylbutyryloxy)-3-oxo-germacran-6*α*,12-olide. The proposed structure was supported by the relevant ^1^H-^1^H COSY, HSQC (Supplementary Materials, Figures S12 and S13), HMBC (Table 3; Supplementary Materials, Figure S14), and NOESY (Supplementary Materials, Figure S15) data as well as by the specific rotation measurement.

Compound **11** was isolated as an amorphous powder. Its HRESIMS spectrum (positive ion mode; Supplementary Materials, Figure S16) showed an adduct ion peak at *m*/*z* 273.1102 [M + Na]^+^ and suggested a molecular formula of C_14_H_18_O_4_Na^+^ (calculated mass: 273.1103). The molecular formula of **11**, established as C_14_H_18_O_4_, denoted six degrees of unsaturation that could be accounted for by two rings, three double bonds, and one carbonyl group (see Figure 3). The proton NMR and ^13^C NMR spectra (Table 4; Supplementary Materials, Figures S17 and S18) together with the ^1^H-^13^C HSQC correlation data (Supplementary Materials, Figure S20) resembled those of eupatobenzofuran [36], except for the signals of the angeloyloxy substituent at C-2 that were replaced with those of the isobutyryl group. The results of the ^1^H-^1^H COSY, HMBC and NOESY experiments (Table 4; Supplementary Materials, Figures S19–S22) supported the assumption that **11** is a new, previously undescribed, structural analog of eupatobenzofuran: 3-hydroxy-3,6-dimethyl-2,3-dihydrobenzofuran-2-yl isobutyrate (carpesibenzofuran).

The known compounds **2**, **3**, **5**–**10**, and **12**–**15** were identified by direct comparison of their spectral data either to those of the compounds isolated earlier in our lab or to those found in the literature [29,30,31,32,33,34,35,37,38,39,40,41,42].

### 2.3. The Cytotoxic and Neuroprotective Activity of 8-Hydroxy-9,10-Diisobutyryloxythymol *(**9**)*

**Figure 4:**
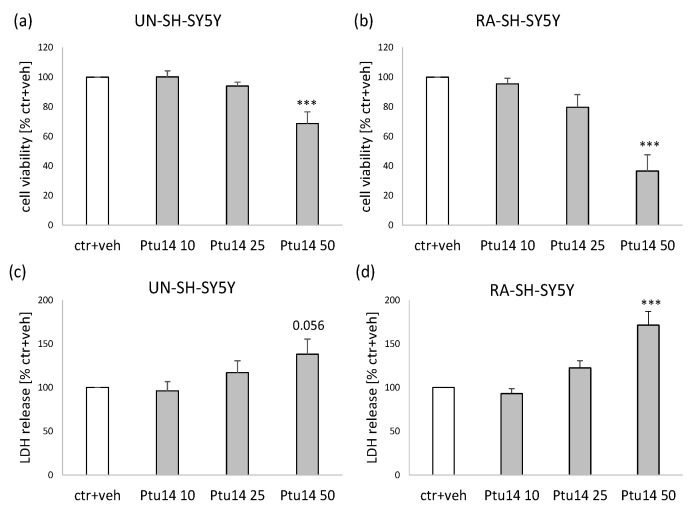
The impact of **9** (Ptu 14) on UN- (**a**,**c**) and RA-SH-SY5Y (**b**,**d**) cell survival after 24 h of treatment. The data from the cell viability MTT reduction assay (**a**,**b**) were normalized to the control (vehicle-treated cells) and are presented as the mean ± SEM from 4–8 independent experiments with 3–5 replicates. Cytotoxicity was measured using the LDH release assay (**c**,**d**) and the data were normalized to the control (vehicle-treated cells) and shown as the mean ± SEM from 4–9 independent experiments with 3–5 replicates. *** *p* < 0.001 vs. vehicle-treated cells.

The thymol derivative (**9**), at a concentration of 50 μM but not 10 or 25 μM, significantly reduced cell viability in undifferentiated (UN-SH-SY5Y) and retinoic acid-differentiated (RA-SH-SY5Y) neuroblastoma cells (by about 30% and 60%, respectively; see Figure 4a,b). At concentrations of 10 and 25 μM, it did not induce any cytotoxic effects in UN- and RA-SH-SY5Y cells, but at a concentration of 50 μM, it significantly increased LDH release (by about 70%) in RA-SH-SY5Y cells. In UN-SH-SY5Y cells, a tendency to increase the level of released LDH (*p* = 0.056, about 40%) was observed (Figure 4c,d). Moreover, based on the MTT assay results, we calculated the IC_50_ for **9** to be 65.7 and 40.9 μM for UN- and RA-SH-SY5Y cells, respectively.

**Figure 5:**
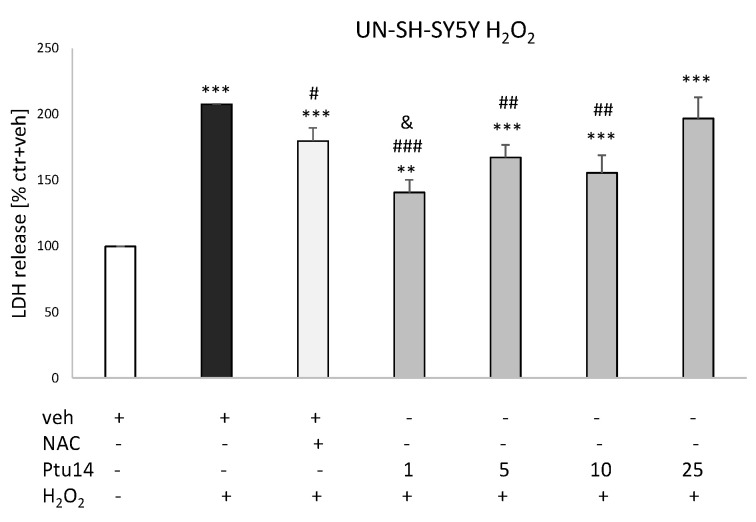
The protective effects of **9** (Ptu14, 1–25 μM) against hydrogen peroxide (H_2_O_2_)-induced cell damage in UN-SH-SY5Y cells measured by the LDH release assay. N-acetyl-cysteine (NAC, 1 mM) was used as the positive control. The data were normalized to the control (vehicle-treated cells) and are presented as the mean ± SEM from 3–5 independent experiments with 3–5 replicates. ** *p* < 0.01 and *** *p* < 0.001 vs. the vehicle-treated cells; # *p* < 0.05, ## *p* < 0.01, and ### *p* < 0.001 vs. the H_2_O_2_-treated cells; and & *p* < 0.05 vs. the NAC + veh + H_2_O_2_-treated cells.

**Figure 6:**
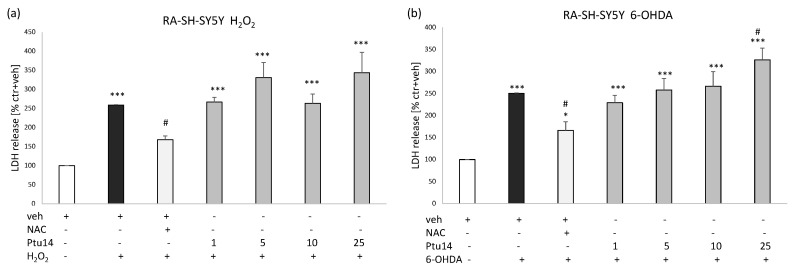
The effect of **9** (Ptu 14; 1–25 μM) on RA-SH-SY5Y cells treated with either H_2_O_2_ (**a**) or 6-hydroxydopamine (**b**). Cell damage effects were measured using the LDH assay. N-acetyl-cysteine (NAC, 1 mM) was used as the positive control. The data were normalized to the control (vehicle-treated cells) and are presented as the mean ± SEM from 4–6 independent experiments with 3–5 replicates. * *p* < 0.05, *** *p* < 0.001 vs. the vehicle-treated cells; ^#^ *p* < 0.05 vs. the H_2_O_2_/6-OHDA-treated cells.

The monoterpenoid (**9**) at concentrations of 1, 5, and 10 μM, but not 25 μM, significantly reduced H_2_O_2_-induced cell damage in the undifferentiated neuroblastoma cells (Figure 5). Moreover, the protective effect of **9** at a concentration of 1 μM was significantly higher than the effect of the positive control, NAC, whereas the other protective concentrations of **9** (5 and 10 μM) demonstrated a similar potency in attenuating H_2_O_2_-induced LDH release as NAC (Figure 5). The compound, however, did not demonstrate any protective activity in RA-SH-SY5Y cells against both H_2_O_2_- and 6-OHDA-induced injury. Nevertheless, we found protective effects of the positive control, NAC, in both models of cell damage. In the 6-OHDA model, at a concentration of 25 μM, **9** significantly increased the cytotoxic effect of this neurotoxin (Figure 6a,b).

## 3. Discussion

Despite the established position of *Carpesium* spp. in traditional Chinese medicine (TCM), the research on the chemical constituents of *C. cernuum* has only been ongoing since the 2000s [4,43]. Germacrane-, eudesmane-, guaiane-, pseudoguaiane-, and xanthane-type sesquiterpene lactones, carabrol, carabrone, mono- and sesquiterpenoids, acyclic diterpenoids, apocarotenoids, sterols, lignans, and flavonoids were identified as metabolites of these species [4,43]. Recently, three new germacranolides, carpescernolides C–E [15], three new acyclic diterpenoids [21] and compositions of the essential oils from the roots and aerial parts of *C. cernuum* [22] have been described. Germacranolides were the most common terpenoids found in *C. cernuum*; however, some of the examined batches of the plant material were either devoid of the compounds or incorrectly botanically identified [44]. So far, except for studies on the essential oil composition, only *C. cernuum* collected in China and Korea has been examined for its phytochemical content.

The plant material grown from the seeds of Romanian origin was rich in germacranolides, especially those of *Carpesium* germacranolide structural type IV [45]. Three of them, incaspitolide D (**7**), **3**, and **8**, were isolated by Liu et al. [18] from *C. cernuum* plants harvested in Guizhou Province (China). The remaining two were divarolide E (**5**), isolated from *Carpesium divaricatum* Sieb. & Zucc. by Zhang et al. [32], and compound **1**, which was described for the first time in this study. Germacranolides **2** and **4** are representatives of structural type III. The former compound was previously described as a constituent of *Inula cuspidata* (DC.) C.B.Clarke [29] and *C. divaricatum* [30], and the latter is a new natural product. Germacranolides of structural types I and II were not isolated from the analyzed plant material, although their presence could not be excluded. Liu et al. [18] found the compounds of all structural types (I–IV) in *C. cernuum*, but the Chinese team started their work with 20 kg of the dry plant material. As was mentioned before [32,33], separation of *Carpesium* germacranolides is a challenge due to their structural similarity and the presence of multiple oxygen functionalities. Although the results of the RP-HPLC-DAD analyses of the fractions eluted from the silica gel column did not show any signals that might represent substantial amounts of the other germacranolides, it cannot be ruled out that these compounds are present in small amounts as the constituents of the multi-component mixtures that were not separated.

The positional isomer of **4**, cardivarolide G, together with two other structurally related germacranolides, was examined earlier for its anti-inflammatory and cytotoxic activity [46]. The compound, in sub-cytotoxic concentrations, exerted significant anti-inflammatory effects on lipopolysaccharide (LPS)-stimulated human neutrophils and induced apoptosis of the osteosarcoma cells independent of the p53 (protein p53) status of the cell line. Cytotoxicity towards different human cancer cell lines in vitro is the most frequently studied activity of the germacranolides isolated from *Carpesium* spp. [15,18,31,32,33,45,46], but its molecular mechanism remains underexplored. Cardivarolide H induces apoptosis in Hep G2 cells and causes cell cycle arrest in the G0/G1 phase [45]. The germacranolides of *C. cernuum* that are active against leukemia cells (HEL, KG-1a, and K562 cells) work via activation of Bcl-2 regulator proteins and induction of cell cycle arrest [15]. Only the cytotoxic actions of incaspitolide A and cernuumolide J have been studied in more detail [13,14,16].

As shown by the results of the TPC estimation, the analyzed plant material was rich in polyphenolic antioxidants. This could not be explained by the yields of lignans and flavonoids that were previously described as the polar constituents of *C. cernuum* [47]. The HPLC-DAD-MS^n^ analysis of the hydroalcoholic (70% MeOH) extracts from the roots and aerial parts of the plant revealed the presence of numerous conjugates of caffeic acid, mainly with quinic and hexaric acids. The conjugates were previously not described as constituents of *C. cernuum*. In particular, caffeoylquinic and caffeoylhexaric acid esters with short-chain organic acids are worth attention. They seem to be the characteristic metabolites of plants from the Inuleae tribe [25,26,27,28], although they can be found in other Asteraceae species as well. The estimated 5-CQA content in the leaves of *C. cernuum* (c. 1%) together with the results of the HPLC-DAD-MS^n^ analysis suggested that hydroxycinnamates are mostly responsible for the antioxidant activity of the plant material.

The chloroform extract from the roots of the examined *C. cernuum* plants contained thymol derivatives (**9**, **13**) and stigmasterol as major constituents. Compound **13** (8,9-epoxy-10-isobutyryloxythymol isobutyrate) was also one of the major constituents of the essential oil distilled from the roots of plants of European origin [22]. The eudesmanolides as well as 3-methyl-8-acetoxy-9,10-diisobutyryloxy-*p*-cymene, 8-hydroxy-9,10-diisobutyryloxythymol 3-methyl ether, and 8-hydroxy-9-acetoxy-10-isobutyryloxythymol 3-methyl ether, which were previously found in the roots of plants collected in China [4,43], seemed to be absent from the material used in this study. The Chinese plant material contained **9** and **13** as minor components. A new natural product, carpesibenzofuran, is a structural analog of eupatobenzofuran, which was isolated from the aerial parts of *Eupatorium cannabinum* L. by Chen and coworkers [36]. It is the first compound of this structural type found in *Carpesium* spp. The absolute configuration of the new compound (**11**) has not been established due to the minute amount of the isolated compound (1.9 mg). The detailed spectral analysis suggested a relative configuration identical to that of eupatobenzofuran. On the other hand, the specific rotation measurement showed a value of 0, suggesting that **11** is a racemic mixture, whereas the value measured for eupatobenzofuran was −7.5.

Thymol derivatives are common constituents of plants from some tribes of Asteraceae, including Inuleae and Eupatorieae. Their biological activity and their molecular mechanisms of action have aroused some interest in recent years. 8-Hydroxy-9,10-diisobutyryloxythymol (**9**) from *Inula wissmanniana* has demonstrated moderate anti-inflammatory activity in LPS-induced RAW 264.7 macrophages [37]. The same compound, isolated from *Inula helianthusaquatilis* C.Y.Wu ex Ling, turned out to be an inhibitor of MDM2-p53 (E3 ubiquitin-protein ligase Mdm2/protein p53) interactions [48]. A search for kinase-inhibiting compounds, which started from 2576 candidate plant extracts, found that 10-isobutyryloxy-8,9-epoxythymyl isobutyrate (**13**) from *Arnica montana* L. is the most active out of all the tested compounds. Compounds **13** and **9** (the possible degradation product of **13**) inhibited aberrant proliferative signaling in melanoma cells via the MAPK/ERK and PI3K/Akt pathways [49]. Moreover, compound **13** from *Inula nervosa* Wall. demonstrated a protective effect in an experimental model of hepatic steatosis [50], in part through the activation of the nuclear factor erythroid 2-related factor 2/antioxidant response element (Nrf2-ARE) signaling pathway.

In the current study, compound **9** was assessed for its neuroprotective and cytotoxic activity in human neuronal-like neuroblastoma SH-SY5Y cells, which are commonly used in neurotoxicity and neuroprotection studies [51,52]. We observed a slightly higher cytotoxicity of **9** in the neuronally differentiated SH-SY5Y cells compared to the undifferentiated ones based on their IC_50_ values. It should be noted that in our previous studies, we observed a higher cytotoxic effect of methyl caffeate or α-rhamnoisorobin (kaempferol 7-*O*-*α*-rhamnopyranoside) in UN- rather than in RA-SH-SY5Y cells [51,52]. This points to the potential utility of **9** at higher concentrations in neuroblastoma treatment for children, especially in the tumor types that are resistant to retinoic acid treatment. However, the antitumor specificity of this effect should be verified in non-transformed cells. Moreover, the mechanism of the cytotoxic action of **9** should be investigated further. In our study, at concentrations up to 25 μM, the compound did not significantly affect the viability of the cells. A similar biosafety profile was demonstrated by α-rhamnoisorobin and methyl caffeate in our previous experiments, which, at concentrations below 50 μM, did not induce cell-damaging effects in UN- or RA-SH-SY5Y cells [51,52]. The partial protective effect of **9** against H_2_O_2_-induced oxidative damage (in the concentration range of 1–10 μM), was only observed in the undifferentiated cells (Figure 5). Compound **9** was not effective in H_2_O_2_- or 6-OHDA-induced oxidative injury of RA-SH-SY5Y cells (Figure 6). It was previously demonstrated that the neuroprotective activity of flavonols (α-rhamnoisorobin, kaempferitrin, and isoquercitrin) is less pronounced in differentiated neuroblastoma cells [52]. During retinoic acid-induced differentiation of neuroblastoma cells, various intracellular pro-survival pathways are activated [53]. This phenomenon could mask or attenuate the neuroprotective action of the studied compounds in the RA-SH-SY5Y cells exposed to 6-OHDA or H_2_O_2_.

## 4. Materials and Methods

### 4.1. Plant Material

This study used *Carpesium cernuum* L. plants grown in the Garden of Medicinal Plants, Maj Institute of Pharmacology PAS in Krakow, from the seeds collected from plants growing in the wild (46°59′2″ N 27°36′13″ E, 345 m.a.s.l.), which were provided by the Anastasie Fătu Botanical Garden of the Alexandru Ioan Cuza University in Iaşi (Romania), as previously described [22]. The roots and aerial parts of the plants were harvested in the beginning of the flowering period (July 2020) in the second year of growth. Voucher specimens (5/18 and 5/19) were deposited in the collection kept at the Garden of Medicinal Plants.

### 4.2. General Methods

Nuclear magnetic resonance (NMR) spectra were recorded either in CDCl_3_ or in CD_3_OD on a Bruker AVANCE III HD 400 (400.17 MHz) spectrometer (Bruker Corp., Billerica, MA, USA). High-resolution mass spectra (HRESIMS) were obtained using a Maldi-SYNAPT G2-S HDMS (Waters Corp., Milford, MA, USA) mass spectrometer equipped with a q-TOF type mass analyzer. Optical rotation was measured in MeOH or CHCl_3_ using a PolAAr31 polarimeter (Optical Activity Ltd., Huntingdon, UK). RP-HPLC-DAD separations were conducted using an Agilent 1200 Series HPLC system (Agilent Technologies Inc., Santa Clara, CA, USA) equipped with a column oven and a diode array detector (DAD). Analytical chromatographic separations were conducted on a Kinetex XB-C18 column (4.6 × 250 mm, total particle size: 5 μm; Phenomenex, Torrance, CA, USA). The preparative RP-HPLC (isocratic mode) was conducted on a Synergi 4μ Fusion-RP, 80A, 250 × 10 mm column (Phenomenex) using MeOH-H_2_O mixtures with different polarities as the eluents. Silica gel 60 (0.063–0.2 mm, Merck, Darmstadt, Germany) and precoated plates (Silica gel 60, Art. No 5553, Merck) were used to perform the conventional column chromatographic separations and TLC separations, respectively.

### 4.3. Materials and Solvents

Analytical-grade organic solvents were purchased either from Avantor Performance Materials S.A. (Gliwice, Poland) or from Merck (Darmstadt, Germany). Water was purified using a Mili-Q system (Milipore Corp., Bedford, MA, USA). HPLC-grade methanol (MeOH) and acetonitrile (MeCN) as well as formic acid and glacial acetic acid were purchased from Merck. Dulbecco’s Modified Eagle’s Medium (DMEM) and fetal bovine serum (FBS) were purchased from Gibco (Invitrogen, Paisley, UK). The Cytotoxicity Detection Kit was supplied by Roche Diagnostic (Mannheim, Germany). The chlorogenic acid standard (5-O-CQA, purity > 97% by HPLC) was purchased from Roth (Karlsruhe, Germany). All other chemicals and reagents were supplied by Sigma-Aldrich Co. (St. Louis, MO, USA).

### 4.4. Total Phenolic Content (TPC) and Chlorogenic Acid (5-CQA) Content Estimation

The reducing capacity of the plant material under study (TPC) was estimated using the Folin–Ciocalteu colorimetric method and the 5-CQA content was assessed using RP-HPLC-DAD, as previously described [27].

### 4.5. Characterization of C. cernuum Hydroalcoholic Extracts Using HPLC-DAD- MS^n^ Method

The plant material (0.1 g) was extracted with 10 mL of 70% (*v*/*v*) MeOH (2 × 3 h) using a rotary shaker. The combined extracts were evaporated *in vacuo* to give a dry residue. A portion (0.01 g) of the residue was dissolved in a mixture of MeOH and 0.1% formic acid (8:2 *v*/*v*), which was then filtered through a 0.45 μm Chromafil membrane (Machery-Nagel, Duren, Germany) and subjected to UHPLC-PAD- MS^n^ analysis. The analysis was performed on a UHPLC-3000 RS system (Dionex, Sunnyvale, CA, USA) with a diode array detector (DAD) and an AmaZon SL ion trap mass spectrometer with an ESI interface (Bruker Daltonics GmbH & Co. KG, Bremen, Germany). Chromatographic separations were conducted using a Zorbax SB-C18 column (150 × 2.1 mm, 1.9 μm; Agilent Technologies Inc., Santa Clara, CA, USA). The column temperature was set to 25 °C. The gradient elution was applied using solvent A (100:0.1 *v*/*v* water–formic acid) and solvent B (100:0.1 *v*/*v* MeCN–formic acid), which were mixed as follows: 0–60 min, 5–40% B. The flow rate was 0.2 mL/min, and the column was equilibrated for 7 min between injections. The UV spectra were recorded over the range of 200–450 nm and the chromatograms were acquired at 325 nm. The LC eluate was introduced, without splitting, directly into the ESI interface. The nebulizer pressure was 40 psi; the dry gas flow was 9 L/min; the dry temperature was 300 °C; and the capillary voltage was 4.5 kV. The mass spectroscopic analysis was conducted using the scan from *m*/*z* 90 to 2200. The compounds were analyzed in the negative ion mode. The MS^2^ fragmentation was obtained for the most abundant ion at the time.

### 4.6. Isolation and Identification of Terpenoids from Roots and Aerial Parts of C. cernuum

The dried and grounded shoots (1 kg) and roots (0.55 kg) of *C. cernuum* were extracted separately with CHCl_3_ (5 × 4.0 L and 5 × 2.0 L, respectively). The organic solvent was evaporated in vacuo to yield 63.3 g and 10.5 g of an oily residue, respectively. The residues were fractionated by conventional CC on silica gel using an n-hexane–EtOAc gradient solvent system (up to 100% EtOAc). The collected fractions (100 mL each for the extract from the aerial parts and 50 mL each for the root extract) were combined according to TLC results. The fractions obtained after the CC separations were further processed using the semi-preparative RP-HPLC technique.

#### 4.6.1. Fractionation of the Extract from *C. cernuum* Aerial Parts

A portion (155 mg) of fractions 101–115 (532 mg), which were eluted with n-hexane–EtOAc (8:2 *v*/*v*), was separated using MeOH-H_2_O (13:7 *v*/*v*) at a flow rate of 1 mL/min to give **1** (17.9 mg) and **2** (8.5 mg). Fractions 135–139 (646 mg) were subjected to CC on silica gel using CHCl_3_ and CHCl_3_-MeOH (99:1 *v*/*v*) as the eluents. The subfraction 135_21 (305 mg), which was obtained from the elution with CHCl_3_-MeOH, was further separated by RP-HPLC (eluent: MeOH-H_2_O (13:7 *v*/*v*); isocratic mode; flow rate: 1.5 mL/min) to yield **3** (12.4 mg), **4** (15.3 mg), and **5** (22.2 mg), plus several mixtures of germacranolides (subtype IV) with different substitution patterns. Fractions 183–191 (452 mg), which were eluted with n-hexane–EtOAc (8:2 *v*/*v*), were subsequently separated by RP-HPLC (eluent: MeOH-H_2_O (3:2 *v*/*v)*; isocratic mode; flow rate: 1.5 mL/min) to give a mixture (30 mg) containing **6** as a major constituent, **7** (74.3 mg), a mixture (1:2, 44.4 mg) of **7** and **8**, and an additional amount of **3** (47.2 mg). Moreover, several mixtures containing subtype IV germacranolides were obtained. Further details of the extraction and fractionation procedure are given in the Supplementary Materials (Figure S23).

#### 4.6.2. Fractionation of the Extract from *C. cernuum* Roots

Fractions 96–103 (183 mg), 120–126 (163 mg), and 131–136 (27 mg), which were eluted with n-hexane–EtOAc (9:1 *v*/*v*), were subsequently separated by RP-HPLC (eluent: MeOH-H_2_O (3:2 *v*/*v*); isocratic mode; flow rate: 1.5 mL/min) to give **9** (99 mg), **10** (19.5 mg), and **11** (1.9 mg). The methanol-insoluble, crystalline part of fractions 96–103 was identified as stigmasterol (**14**). Fractions 185–193 (109 mg) and 202–204 (19 mg), which were eluted with n-hexane–EtOAc (7:3 *v*/*v*), were further subfractionated by RP-HPLC (eluent: MeOH-H_2_O (1:1 *v*/*v*); isocratic mode; flow rate: 1.5 mL/min) to yield **12** (8.3 mg) and **15** (1.7 mg). For further details, see the Supplementary Materials (Figure S24).

A freshly prepared extract from the roots of *C. cernuum* was examined using RP-HPLC-DAD and ^1^H NMR analyses (Supplementary Materials, Figure S1) and was shown to contain 8,9-epoxy-10-isobutyryloxythymol isobutyrate (**13**) as the major constituent. The compound was eluted from the silica gel column with n-hexane-EtOAc (9:1 *v*/*v*) and was a component of fractions 81–93 that were not processed further.

#### 4.6.3. Characterization of Compounds **1**, **4**, and **11**

*8α-angeloyloxy-4β-hydroxy-5β-(3-methylbutyryloxy)-9-oxo-germacran-6α,12-olide* (**1**). White amorphous solid: [α]_D_^25^.^5^: + 62.3° (c = 0.2, MeOH); UV (MeCN-H_2_O) λ_max_ 216 nm; ^1^H- and ^13^C-NMR: Table 2; 1D and 2D NMR: Supplementary Materials Figures S3–S8; HRESIMS (pos. mode) *m*/*z* 487.2304 [M + Na]^+^, calc. 487.2308, Supplementary Materials Figure S2.

*9β-angeloyloxy-4β,8α-dihydroxy-5β-(3-methylbutyryloxy)-3-oxo-germacran-6α,12-olide* (**4**). Colorless needles: [α]_D_^25^.^5^:−40° (c = 0.2, MeOH); UV (MeCN-H_2_O) λ_max_ 214 nm; ^1^H- and ^13^C-NMR: Table 3; 1D and 2D NMR: Supplementary Materials Figures S10–S15; HRESIMS (neg. mode) *m*/*z* 479.2282 [M–H]^−^, calc. 479.2281, Supplementary Materials Figure S9.

*3-hydroxy-3,6-dimethyl-2,3-dihydrobenzofuran-2-yl isobutyrate* (carpesibenzofuran, **11**). Colorless amorphous solid: [α]_D_^25^.^5^: 0° (c = 0.2, CDCl_3_); UV (MeCN-H_2_O) λ_max_ 277 nm; ^1^H- and ^13^C-NMR: Table 4; 1D and 2D NMR: Supplementary Materials Figures S17–S22; HRESIMS (pos. mode) *m*/*z* 273.1102 [M + Na]^+^, calc. 273.1103, Supplementary Materials Figure S16.

The structures of the compounds were determined based on their spectral data (HRESIMS, 1D and 2D NMR, and specific rotation) with reference to those of compounds that were previously isolated in our lab and available literature data.

### 4.7. SH-SY5Y Cell Culture

The human neuroblastoma SH-SY5Y cell line was obtained from the American Type Culture Collection (CRL-2266, ATCC, Manassas, VA, USA) and the cells were cultured as described previously [54]. Confluent cells (c. 80%) were counted using a Bürker chamber and seeded into 96-well plates at a density of 3 ×10^4^ cells per well. The differentiation of the cells to the neuronal phenotype was induced as described in [54]. One day prior to the experiments, the culture media for both cell phenotypes (UN-SH-SY5Y and RA-SH-SY5Y) were replaced with DMEM supplemented with 1% FBS and a 1% penicillin/streptomycin solution to limit cell proliferation. The cells used for the experiments were from passages 4–15.

### 4.8. Cell Treatment

To assess the biosafety and putative cytotoxic effects of **9** in UN-SH-SY5Y and RA-SH-SY5Y, the cells were treated for 24 h with **9** (10, 25, and 50 μM) in the experimental cell culture medium (1% FBS). For the assessment of neuroprotective activity, the UN- and RA-SH-SY5Y cells were pretreated with **9** (30 min; 1, 5, 10, and 25 μM) followed by a 24 h exposure to H_2_O_2_ (0.375 mM and 0.5 mM for UN- and RA-SH-SY5Y cells, respectively). Moreover, we evaluated the neuroprotective effect of the compound in a model of RA-SH-SY5Y cell damage induced by 6-hydroxydopamine (6-OHDA; 200 μM). The effective concentrations of H_2_O_2_ and 6-OHDA were established in our previous studies [51]. As a positive control, the antioxidant N-acetyl-cysteine (NAC, 1 mM) was used. The compound was applied concomitantly with H_2_O_2_ or 6-OHDA, as described previously [51]. The stock solutions of **9** (5, 2.5, 1, 0.5, and 0.1 mM) were prepared using 70% ethanol and stored at −20 °C. The stock solution of NAC (100 mM) was prepared in sterile distilled water and stored at −20 °C. The H_2_O_2_ stock solutions (25 and 50 mM) in distilled water were prepared from stabilized 30% hydrogen peroxide just before the experiments. The 20 mM 6-OHDA stock solution was made with distilled water immediately before use. All agents were added to the culture medium at the indicated concentrations under light limited conditions. Each set of control cultures was supplemented with the corresponding vehicles, and the final concentration of the solvent in the experimental cultures was limited to 1%.

### 4.9. Cell Viability (MTT) and Cytotoxicity (LDH Release) Assays

The effect of **9** on the cell viability of neuroblastoma cells was measured using the 3-[4,5-dimethylthiazol-2-yl]-2,5-diphenyltetrazolium bromide (MTT) assay, as described previously [53]. The absorbance of the probes was measured at 570 nm with an Infinite M200 PRO microplate reader (Tecan GmbH, Grödig, Austria). For the cytotoxicity and neuroprotection assessments, the LDH assay was used (Cytotoxicity Detection Kit, Roche Diagnostic) following the supplier’s instruction [51]. The absorbance of the probes was measured at 490 nm using the same plate reader used for the MTT test.

### 4.10. Statistics

The biological data from 3–9 independent experiments after normalization to the control (vehicle-treated cells, set as 100%) were statistically analyzed using one-way analysis of variance (one-way ANOVA) and post hoc Duncan tests for multiple comparisons using the Statistica 13 software (StatSoft Inc., Tulsa, OK, USA). *p* < 0.05 was considered statistically significant. The IC_50_ values were calculated from the results of the MTT test (after 24 h of treatment with **9**) using GraphPad Prism 5 (GraphPad Software Inc., San Diego, CA, USA) and the nonlinear regression ‘log (inhibitor) vs. normalized response—Variable slope’ option.

## 5. Conclusions

*C. cernuum* plants of European origin are rich in hydroxycinnamates, which are structurally related to those found in other Inuleae species. The compounds may be mainly responsible for the high reducing capacity of the plant extract. Two new germacranolides (structural types III and IV) were found in the aerial parts of the *C. cernuum* plants. Structural type I and II compounds were not isolated but they might be present in the analyzed plant material as minor constituents. The roots of the plants yielded known thymol derivatives and a new natural product, carpesibenzofuran, a structural analog of eupatobenzofuran. One of the thymol derivatives isolated from the *C. cernuum* roots, 8-hydroxy-9,10-diisobutyryloxythymol, exerted a limited protective effect against hydrogen peroxide-induced damage in undifferentiated neuroblastoma cells at concentrations of 1–10 μM. In summary, European *C. cernuum* plants are rich in biologically active metabolites and their biochemistry needs further study. Terpenoids isolated from the plant material could be further examined to assess their biological activity profile.

## Figures and Tables

**Table 1:** Retention times, UV maxima, and MS^n^ data (in the negative ion mode) for the compounds present in the hydroalcoholic (70% MeOH) extracts from *Carpesium cernuum* L.

	Compound	t_R_ [min]	UV [nm]	[M−H]^−^ *m*/*z*	Productions Main Peak(s) *m*/*z* ^1^	Roots	Aerial Parts
**1**	Caffeoylhexaric acid (I)	5.2	325	371	353, **209**, 191	+	−
**2**	Caffeoylhexaric acid (II)	7.7	325	371	353, **209**, 191	+	−
**3**	Caffeoylhexaric acid (III)	9.7	325	371	353, **209**, 191	+	−
**4**	Caffeoylhexaric acid (IV)	12.5	325	371	353, **209**, 191	+	−
**5**	5-*O*-Caffeoylquinic acid	17.3	325	353	**191**	+	+
**6**	Dicaffeoylhexaric acid (I)	21.4	323	533	353, **371**, 209, 191	+	+
**7**	Dicaffeoylhexaric acid (II)	23.8	326	533	353, **371**, 209, 191	+	+
**8**	Dicaffeoylhexaric acid (III)	27.6	327	533	353, **371**, 209, 191	+	+
**9**	Unidentified caffeoylglucose derivative	28.3	319	509	**428**, **341**, 323, 179	+	+
**10**	Dicaffeoylhexaric acid (IV)	28.9	327	533	353, **371**, 209, 191	+	+
**11**	3,4-Di-*O*-caffeoylquinic acid	33.1	325	515	**353**, 335, 299, 255, 203, 179, 173	−	+
**12**	1,5-Di-*O*-caffeoylquinic acid	34.6	328	515	**353**, 335, 191	+	+
**13**	3,5-Di-*O*-caffeoylquinic acid	34.7	327	515	**353**, 191, 179	−	+
**14**	Tricaffeoylhexaric acid (I)	35.6	327	695	**533**, 371, 209	+	+
**15**	4,5-Di-O-caffeoylquinic acid	37.0	327	515	**353**, 317, 299, 255, 203, 191, 179,173	−	+
**16**	Tricaffeoylhexaric acid (II)	38.5	328	695	**533**, **371**, 353, 209	+	+
**17**	Isobutyryl-dicaffeoylhexaric acid	43.4	328	603	**441**, 423, 353, 335, 279, 191	+	−
**18**	Isobutyryl-tetracaffeoylhexaric acid	46.0	324	927	**765**, **603**, 441, 423, 341	+	−
**19**	Tetracaffeoylhexaric acid	46.6	329	857	**695**, 533	+	+
**20**	Isobutyryl-tricaffeoylhexaric acid (I)	51.6	328	765	**603**, **441**, 423, 353, 279	+	+
**21**	Isobutyryl-tricaffeoylhexaric acid (II)	53.3	328	765	**603**, **441**, 423, 353, 279	+	−
**22**	Isobutyryl-tricaffeoylhexaric acid (III)	54.4	328	765	**603**, **441**, 423, 353, 279	+	−
**23**	2-Methylbutyryl/isovaleryl-tricaffeoylhexaric acid (I)	55.4	328	779	**617**, **445**, 353, 293, 191	+	+
**24**	2-Methylbutyryl/isovaleryl-tricaffeoylhexaric acid (II)	55.7	328	779	**617**, **445**, 353, 293, 191	+	+

^1^ Ions in bold are the most abundant ion peaks; +: detected in the extract; −: not detected in the extract.

**Table 2:** ^1^H NMR (400.17 MHz) data of compound **1** in both CDCl_3_ and CD_3_OD and ^13^C NMR (100.63 MHz) data of **1** in CD_3_OD.

Position	δ_H_ (ppm), *J* (Hz) (CDCl_3_)	δ_H_ (ppm), *J* (Hz) (CD_3_OD)	δ_C_ (ppm)	HMBC (H → C)
1	1.47–1.80 m ^b^	1.26 m, 1.71 m	21.4	C-2, C-10, C-14
2	1.47–1.80 m ^b^	1.48 m, 1.57 m	36.2	C-1, C-3
3	1.47–1.80 m ^b^	1.70 m, 1.70 m	33.1	C-1, C-2
4	-	-	72.4	-
5	4.64 d (6.1)	4.72 d (6.3)	77.3	C-6, C-7, C-15, C-1′
6	4.68 dd (6.1, 1.7)	4.63 dd (6.3, 1.6)	72.0	C-4, C-5, C-7, C-8, C-11, C-12
7	3.50 br d (11.3)	3.87 dd (11.3, 1.6)	44.9	C-5, C-8, C-11, C-12
8	4.99 d (11.3)	4.95 d (11.3)	78.4	C-6, C-7, C-10, C-11, C-1”
9	-	-	212.1	-
10	3.07 m	3.31 m	41.3	C-3, C-14
11	-	-	133.5	-
12	-	-	169.4	-
13	6.38 s 5.90 s	6.29 d (1.5) 5.98 d (1.2)	126.0	C-7, C-8, C-11, C-12
14	1.04 d (6.7)	1.03 d (6.7)	19.5	C-1, C-10
15	1.14 s	1.16 s	23.5	C-3, C-4, C-5
1′	-	-	173.4	-
2′	2.31 m	2.30 m	42.2	C-1′, C-3′, C-4′, C-5′
3′	2.10 m	2.10 m	25.1	C-2′, C4′, C-5′
4′	0.96 d (6.3) ^a^	0.97 d (6.6)	21.4	C-2′, C-3′, C-5′
5′	0.97 d (6.3) ^a^	0.97 d (6.6)	21.4	C-2′, C-3′, C-4′
1”	-	-	165.7	-
2”	-	-	126.0	-
3”	6.24 qq (7.2, 1.0)	6.33 m	141.7	C-4”, C-5”
4”	1.97 brs	2.01 d (1.4) ^b^	19.2	-
5”	2.01 dq (7.2, 1.0)	2.03 dq (7.2, 1.4) ^b^	14.7	C-3”

^a^ Interchangeable signals; ^b^ overlapping signals.

**Table 3:** ^1^H NMR (400.17 MHz) and ^13^C NMR (100.63 MHz) data of compound **4** in CD_3_OD.

Position	δ_H_ (ppm), *J* (Hz)	δ_C_ (ppm)	HMBC (H → C)
1	1.88 m, 1.74 m	25.4	
2	3.85 m, 2.24 m ^b^	33.0	
3	-	217.7	
4	-	80.4	
5	5.41 d (11.6)	78.2	C-6, C-1′
6	4.67 dd (11.6, 6.4)	79.9	C-5, C-8
7	3.05 m	41.7	C-5
8	4.45 d (10.2)	70.6	
9	5.26 d (10.2)	78.5	C-8, C-1′′
10	2.24 m ^b^	30.0	
11	-	132.8	
12	-	169.6	
13	6.34 d (3.0), 5.70 d (2.5)	123.9	C-7, C-12
14	0.98 d (6.9)	20.0	C-1, C-9, C-10
15	1.24 s	23.5	C-4, C-5
1′	-	172.4	
2′	2.35 m	42.8	C-1′, C-3′, C-4′, C-5′
3′	2.16 m	25.4	C-1′, C-4′, C-5′
4′	1.01 d (6.6) ^a^	21.4	C-2′, C-3′, C-5′
5′	1.02 d (6.6) ^a^	21.4	C-2′, C-3′, C-4′
1″	-	167.8	
2″	-	127.9	
3″	6.16 qq (7.2, 1.4)	137.7	
4″	1.95 q (1.4)	19.5	C-1″, C-2″, C-3″
5″	1.99 dq (7.2, 1.4)	14.7	C-1″, C-2″, C-3″

^a^ Interchangeable signals; ^b^ overlapping signals.

**Table 4:** ^1^H NMR (400.17 MHz) and ^13^C NMR (100.63 MHz) data of compound **11** in CDCl_3_.

Position	δ_H_ (ppm), *J* (Hz)	δ_C_ (ppm)	HMBC (H → C)
2	6.52 s	104.9	C-1′, C-7a
3	-	79.8	-
4	7.24 d (7.6)	122.7	C-6, C-7a
5	6.87 br d (7.6)	123.1	C-3a
6	-	141.0	-
7	6.78 brs	111.7	C-5
3a	-	127.6	-
7a	-	158.5	-
Me-3	1.65 s	20.1	C-2, C-3, C-3a
Me-6	2.37 s	21.7	C-5, C-6, C-7, C-7a
1′	-	175.7	-
2′	2.61 m (7.0)	34.1	C-1′
3′	1.22 d (6.8) ^a^	18.8 ^a^	C-1′, C-2′, C-4′
4′	1.20 d (6.8) ^a^	18.5 ^a^	C-1′, C-2′, C-3′

^a^ Interchangeable signals.

## Data Availability

Data is contained within the article and Supplementary Materials.

## Supplementary Materials

The following supporting information can be downloaded at: https://www.mdpi.com/article/10.3390/molecules30122506/s1. ^1^H NMR spectrum of a chloroform extract from roots of *Carpesium cernuum* together with proton NMR spectrum of **13** (Figure S1); MS and NMR spectra of compounds **1**, **4**, and **11** (Figures S2–S22, Table S1); fractionation schemes of chloroform extracts from aerial parts and roots of *C. cernuum* (Figures S23 and S24).
