# Supplementary material for: New Terpenoids and Polyphenolic Profile of Carpesium cernuum L. of European Origin
Source: Molecules. 2025 Jun 7;30(12):2506. doi: 10.3390/molecules30122506 (PMC12195868; doi:10.3390/molecules30122506)
Supplement: Supplementary file 1 [file molecules-30-02506-s001.zip › molecules-3645638-supplementary.pdf]

SUPPLEMENTARY MATERIAL

New terpenoids and polyphenolic profile of *Carpesium cernuum* L. of European origin

Janusz Malarz 1, Danuta Jantas 1, Klaudia Jakubowska 1, Ryszard Bugno 1, Anna Karolina Kiss 2 and Anna Stojakowska 1*

[1] Maj Institute of Pharmacology, Polish Academy of Sciences, Smętna street 12, 31-343 Kraków, Poland; malarzj@if-pan.krakow.pl; jantas@if-pan.krakow.pl; bugno@if-pan.krakow.pl; stoja@if-pan.krakow.pl
[2] Department of Pharmaceutical Biology, Medical University of Warsaw, Banacha street 1, 02-097 Warsaw, Poland; akiss@wum.edu.pl
* Correspondence: stoja@if-pan.krakow.pl; Tel.: +481-26-623-254 (A.S.)

Figure S1: ^{1}H NMR spectrum of a chloroform extract from roots of *Carpesium cernuum* (upper part) and ^{1}H NMR spectrum of a standard sample of 8.9-epoxy-10-isobutyryloxythymol isobutyrate (compound **13**; lower part)

Figure S2: HRESIMS spectrum of compound **1.**

Figure S3: ^{1}H NMR spectrum of compound **1** in $CDCl_3$ (upper part) and CD_3OD (lower part).

Figure S4: ^{13}C NMR spectrum of compound **1** in CD_3OD.

Figure S5: COSY spectrum of compound **1** in CD_3OD.

Figure S6: HSQC spectrum of compound **1** in CD_3OD.

Figure S7: HMBC spectrum of compound **1** in CD_3OD.

Figure S8: NOESY spectrum of compound **1** in CD_3OD.

Figure S9: HRESIMS spectrum of compound **4**.

Figure S10: ^{1}H NMR spectrum of compound **4** in CD_3OD.

Figure S11: ^{13}C NMR spectrum of compound **4** in CD_3OD.

Figure S12: COSY spectrum of compound **4** in CD_3OD.

Figure S13: HSQC spectrum of compound **4** in CD_3OD.

Figure S14: HMBC spectrum of compound **4** in CD_3OD.

Figure S15: NOESY spectrum of compound **4** in CD_3OD.

Figure S16: HRESIMS spectrum of compound **11**.

Figure S17: ^{1}H NMR spectrum of compound **11** in $CDCl_3$.

Figure S18: ^{13}C NMR spectrum of compound **11** in $CDCl_3$.

Figure S19: COSY spectrum of compound **11** in $CDCl_3$.

Figure S20: HSQC spectrum of compound **11** in $CDCl_3$.

Figure S21: HMBC spectrum of compound **11** in $CDCl_3$.

Figure S22: NOESY spectrum of compound **11** in $CDCl_3$.

Table S1. ^{13}C NMR (100.63 MHz) data of 8*α*-angeloyloxy-4*β*-hydroxy-5*β*-(3-methylbutyryloxy)-9-oxo-germacran-7*β*,12-olide (**1**), 8*α*-angeloyloxy-4*β*-hydroxy-5*β*-isobutyryloxy-9-oxo-germacran-7*β*,12-olide [30], 9*β*-angeloyloxy-4*β*,8*α*-dihydroxy-5*β*-(3-methylbutyryloxy)-3-oxo-germacran-6*α*,12-olide (**4**), and cardivarolide G [33] in CD_3OD.

Figure S23: Fractionation scheme: A flowchart representing the chromatographic separation of a crude chloroform extract from aerial parts of *Carpesium cernuum* L. (EtOAc: ethyl acetate; MeOH: methanol; conditions for the preparative HPLC separations are specified in section 4.6.1. of the main text).

Figure S24: Fractionation scheme: A flowchart representing the chromatographic separation of a crude chloroform extract from roots of *Carpesium cernuum* L. (EtOAc: ethyl acetate; MeOH: methanol; conditions for the preparative HPLC separations are specified in section 4.6.2. of the main text).

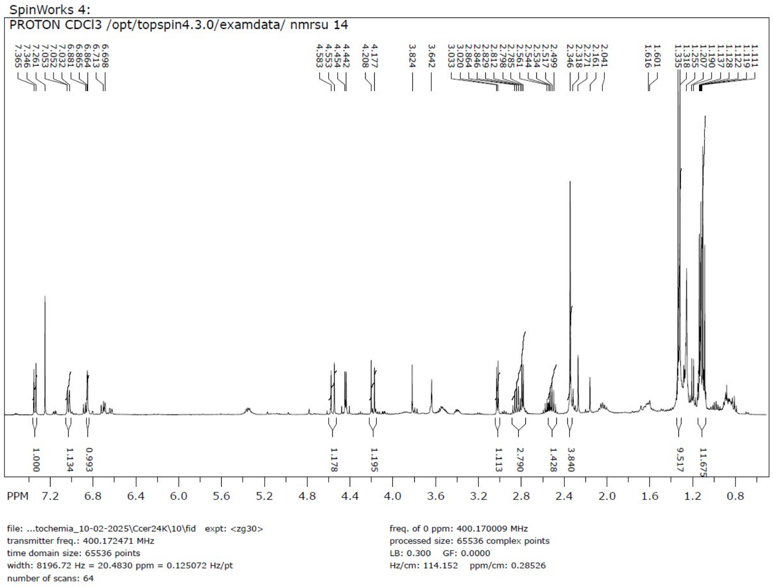

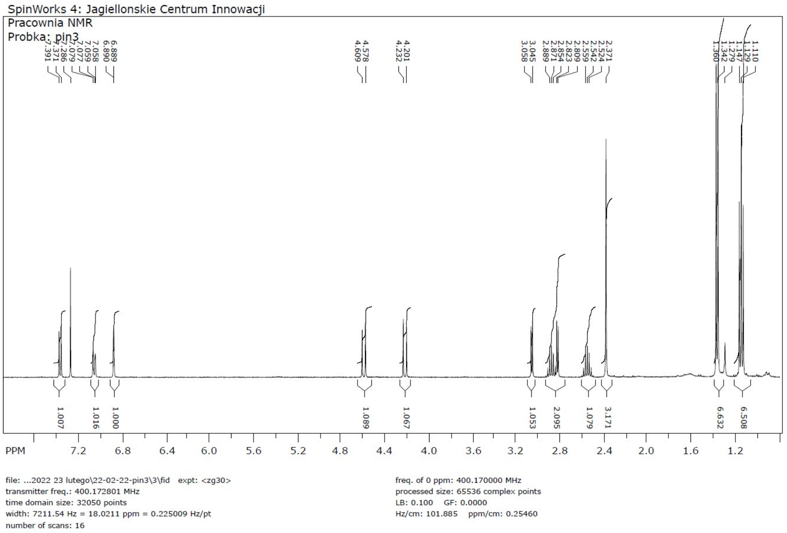

Figure S1: ^{1}H NMR spectrum of a chloroform extract from roots of *Carpesium cernuum* (upper part) and ^{1}H NMR spectrum of a standard sample of 8.9-epoxy-10-isobutyryloxythymol isobutyrate (compound **13**; lower part)

Single Mass Analysis
Tolerance = 3.0 PPM / DBE: min = -1.5, max = 50.0
Element prediction: Off
Number of isotope peaks used for i-FIT = 3
Monoisotopic Mass, Even Electron Ions
10 formula(e) evaluated with 1 results within limits (up to 50 closest results for each mass)
Elements Used:
C: 0-200 H: 0-200 O: 7-8 Na: 1-1

Mass	Calc. Mass	mDa	PPM	DBE	Formula	i-FIT	i-FIT Norm	Fit Conf %	C	H	O	Na
487.2304	487.2308	-0.4	-0.8	7.5	C25 H36 O8 Na	1074.4	n/a	n/a	25	36	8	1

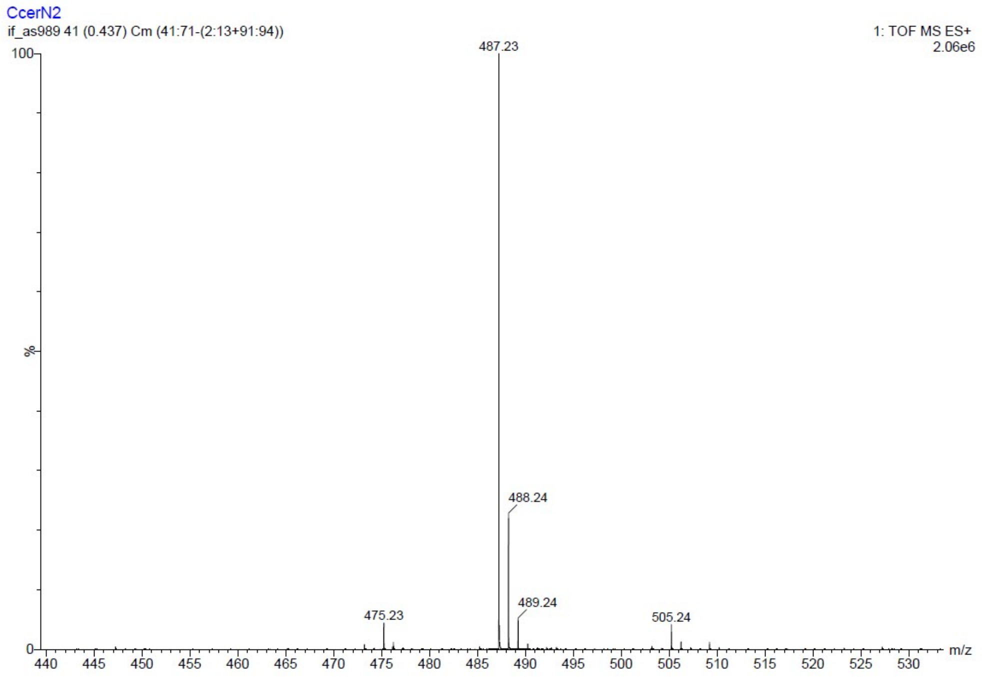

Figure S2: HRESIMS spectrum of compound **1**

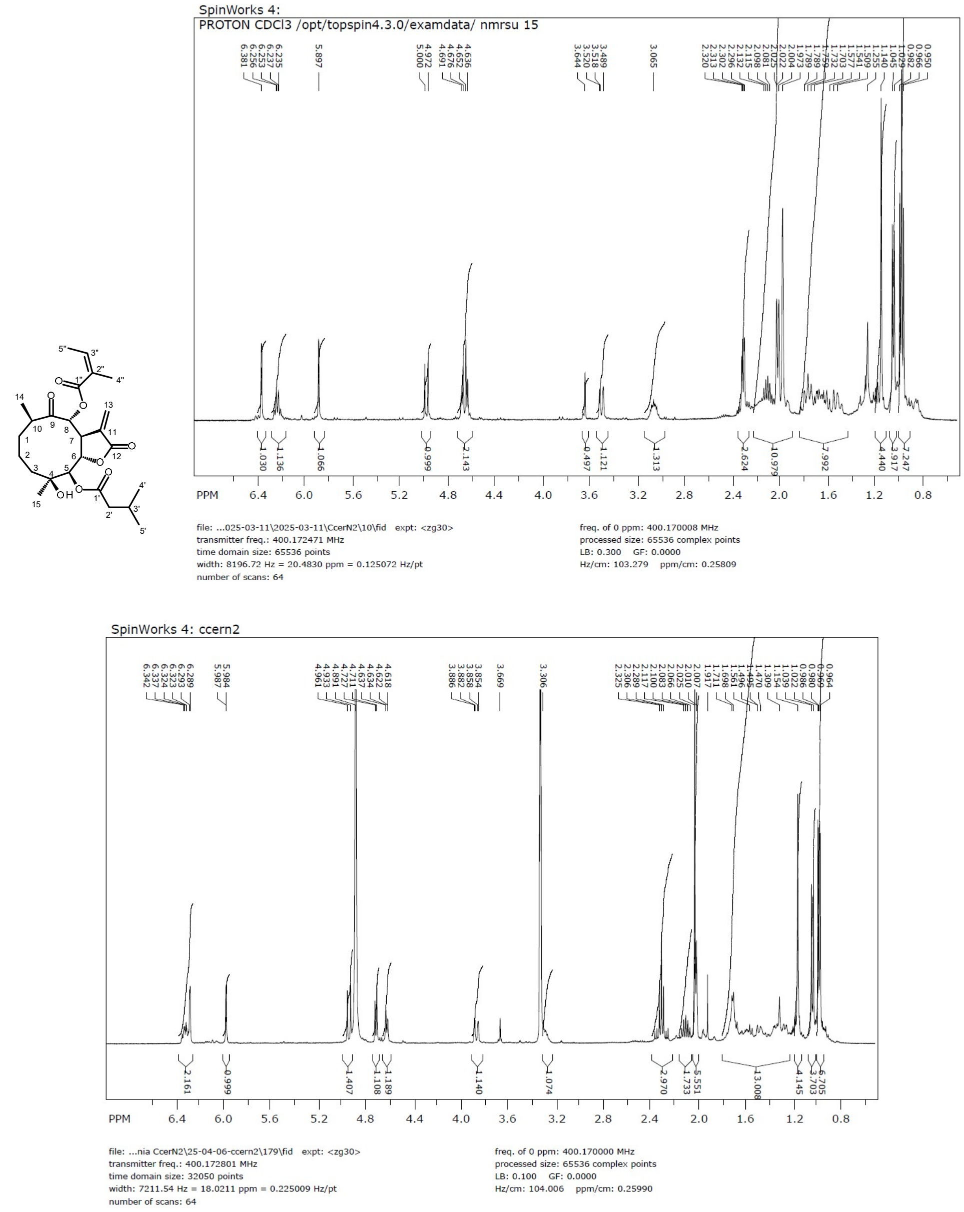

Figure S3: ^{1}H NMR spectrum of compound **1** in $CDCl_3$ (upper part) and CD_3OD (lower part).

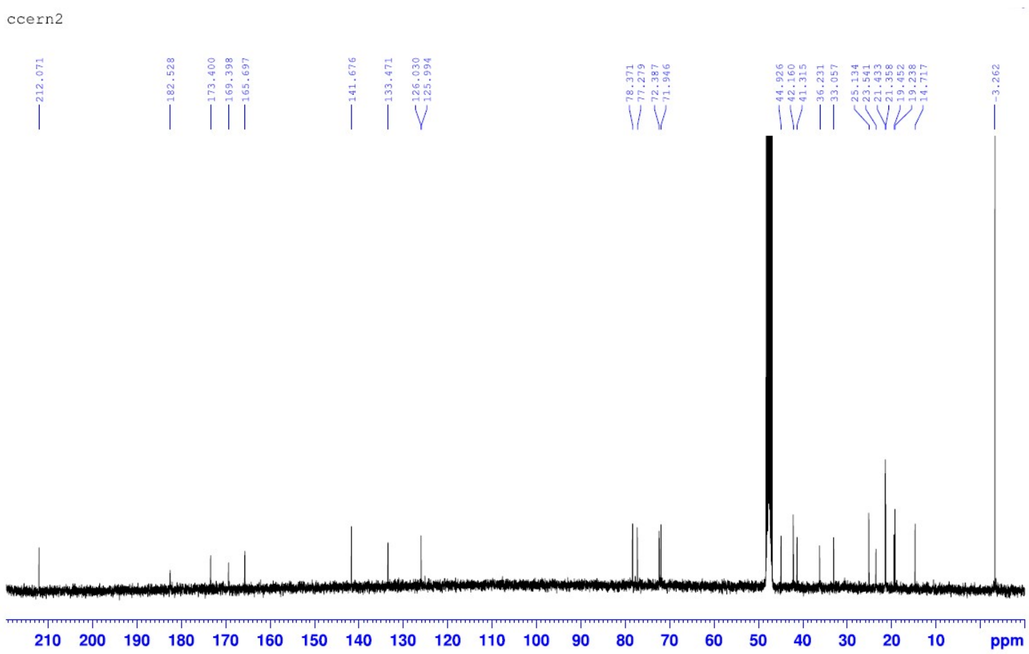

Figure S4: ^{13}C NMR spectrum of compound **1** in CD_3OD.

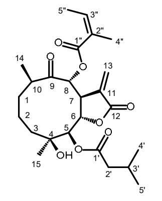

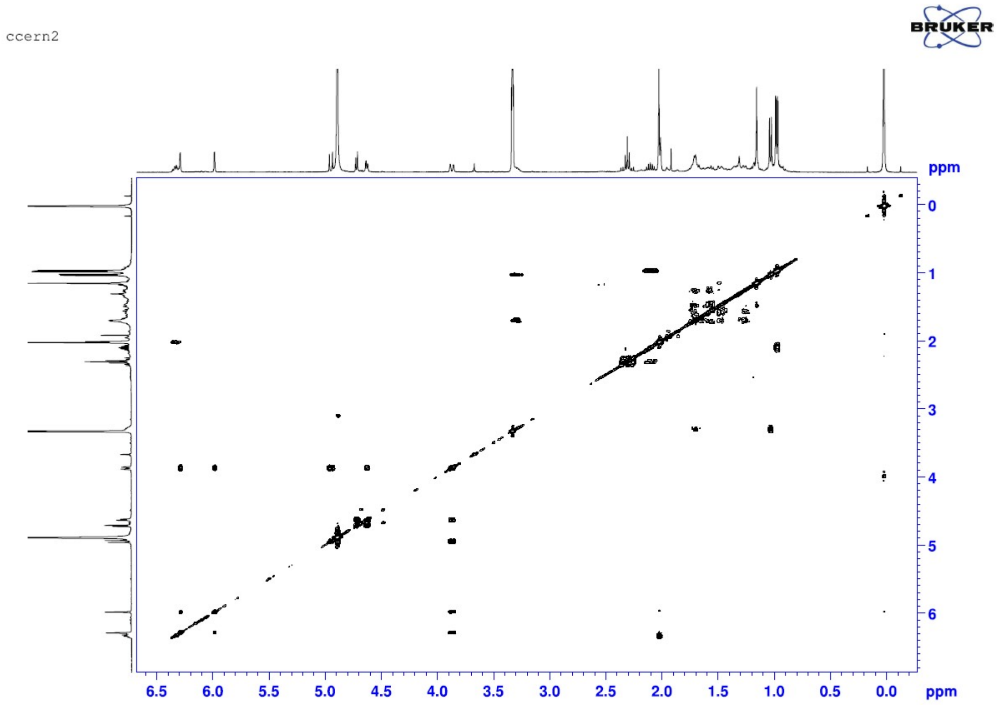

Figure S5: COSY spectrum of compound **1** in CD_3OD.

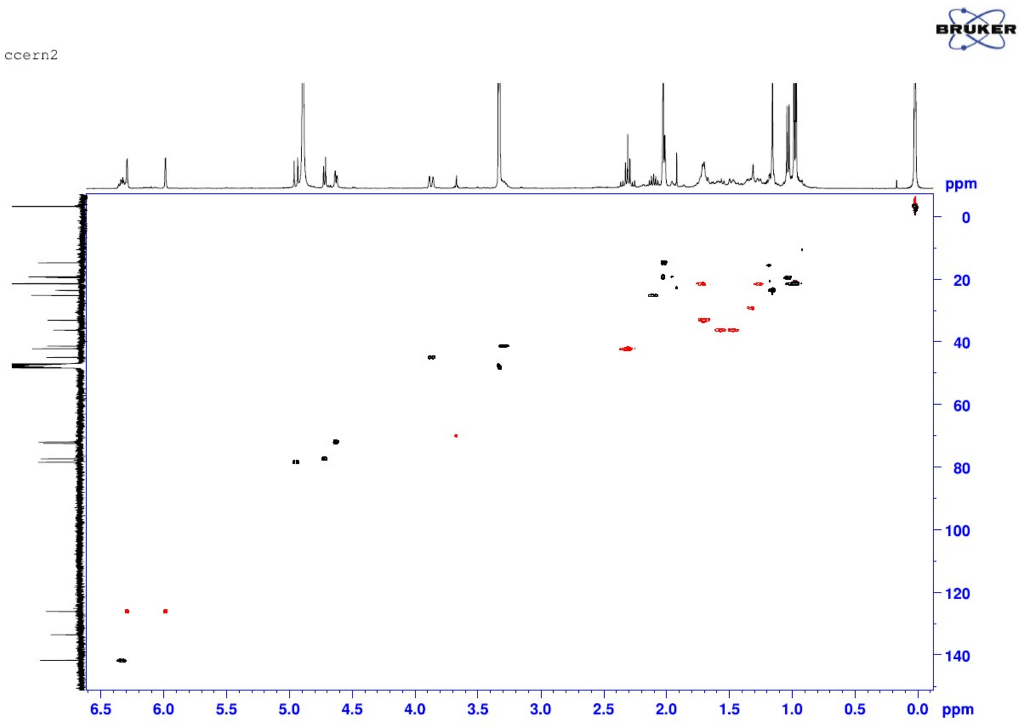

Figure S6: HSQC spectrum of compound **1** in CD_3OD.

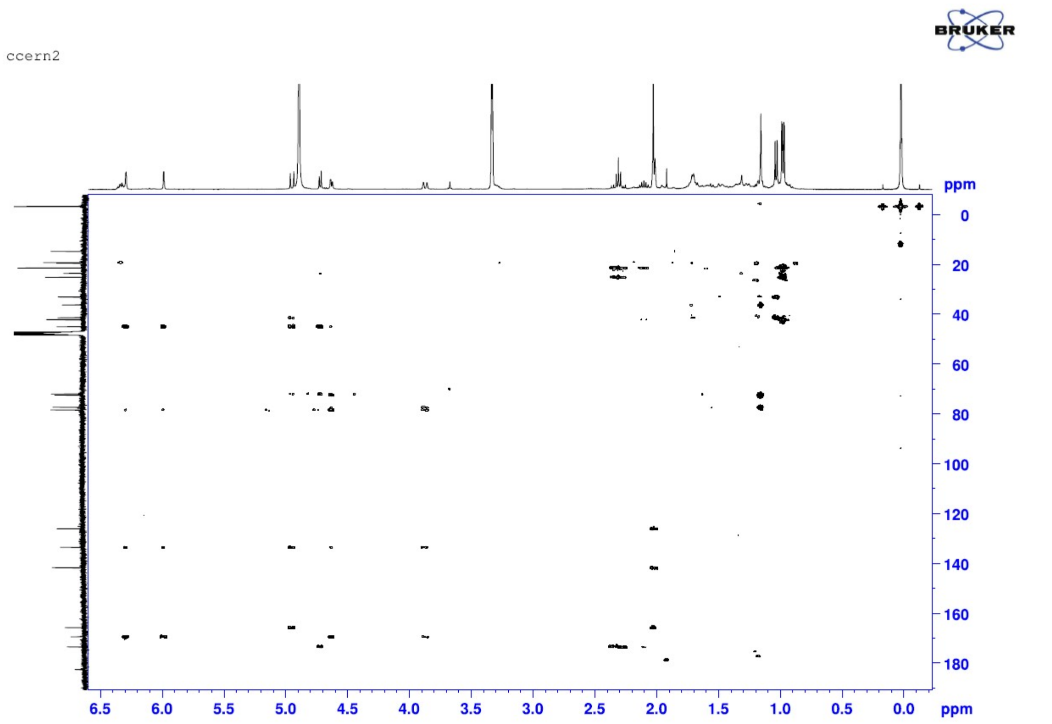

Figure S7: HMBC spectrum of compound **1** in CD_3OD.

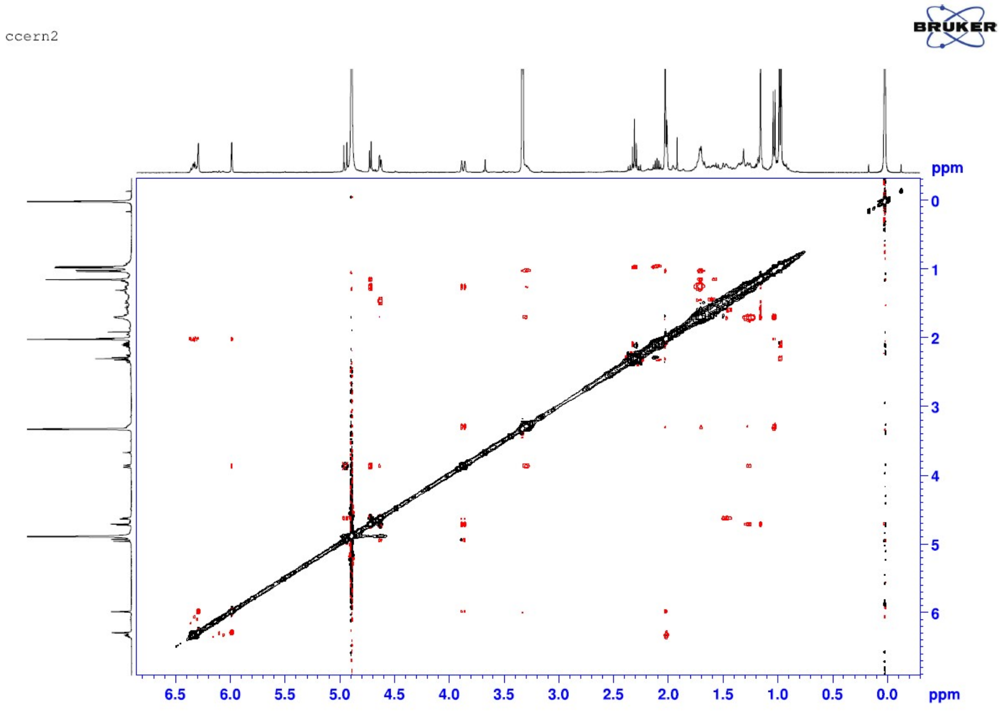

Figure S8: NOESY spectrum of compound **1** in CD_3OD.

Single Mass Analysis
Tolerance = 3.0 PPM / DBE: min = -1.5, max = 50.0
Element prediction: Off
Number of isotope peaks used for i-FIT = 3
Monoisotopic Mass, Even Electron Ions
15 formula(e) evaluated with 1 results within limits (up to 50 closest results for each mass)
Elements Used:
C: 0-200 H: 0-200 O: 7-9

Mass	Calc. Mass	mDa	PPM	DBE	Formula	i-FIT	i-FIT Norm	Fit Conf %	C	H	O
479.2282	479.2281	0.1	0.2	8.5	C25 H35 O9	1262.8	n/a	n/a	25	35	9

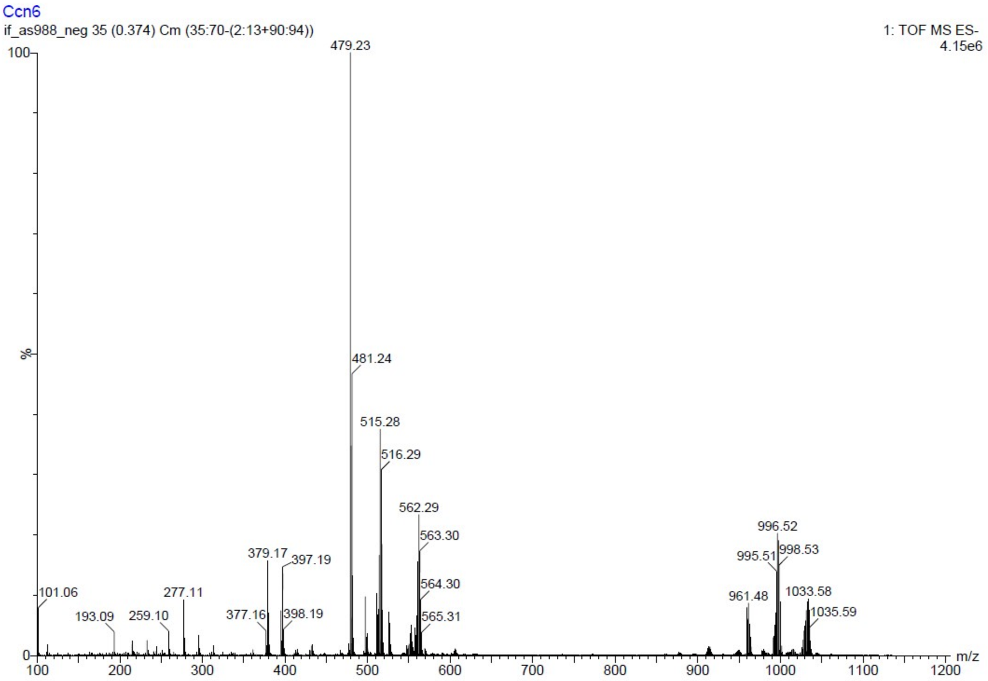

Figure S9: HRESIMS spectrum of compound **4**.

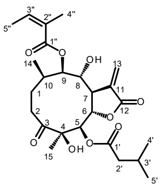

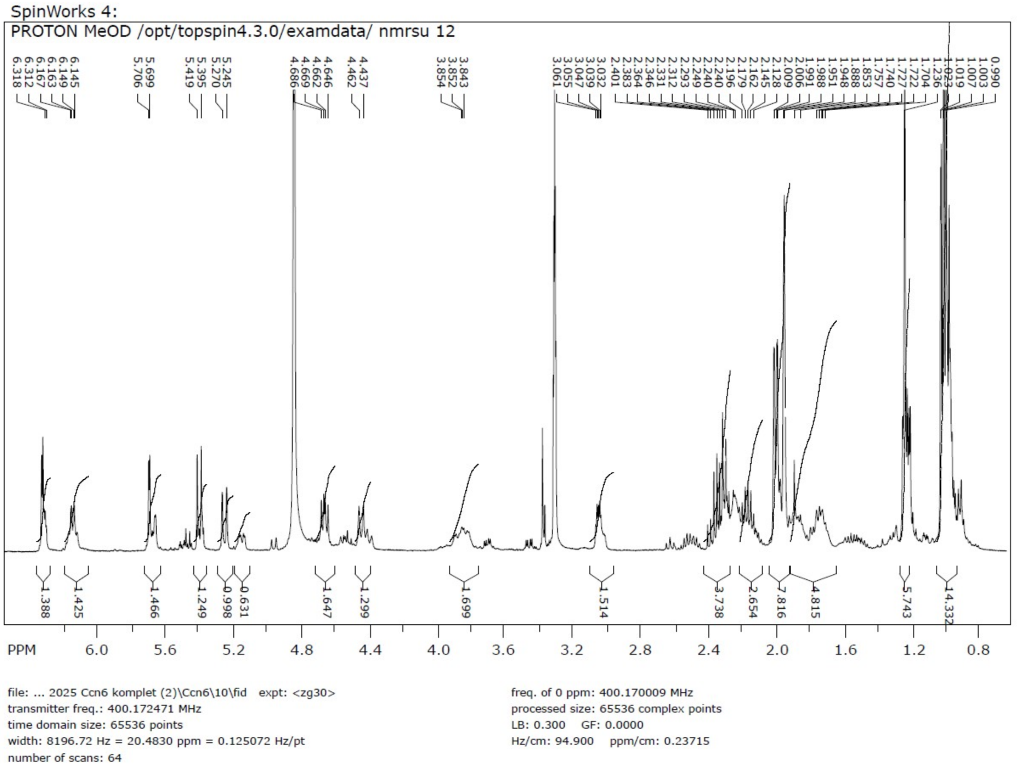

Figure S10: ^{1}H NMR spectrum of compound **4** in CD_3OD.

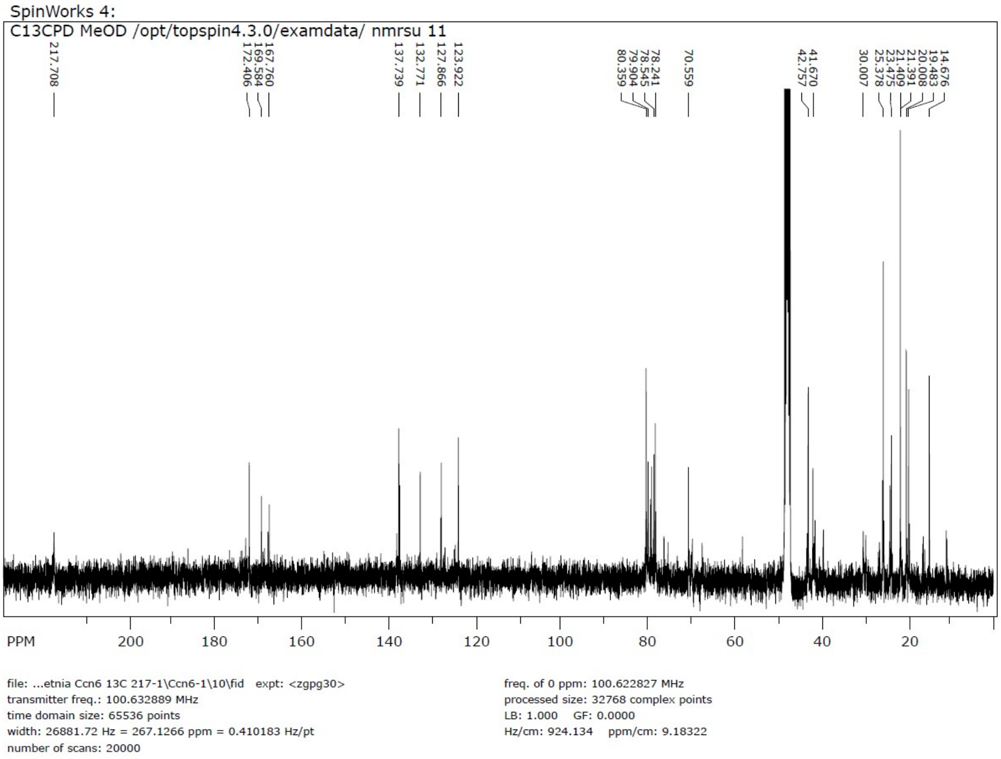

Figure S11: ^{13}C NMR spectrum of compound **4** in CD_3OD.

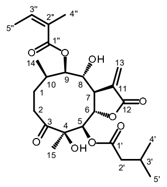

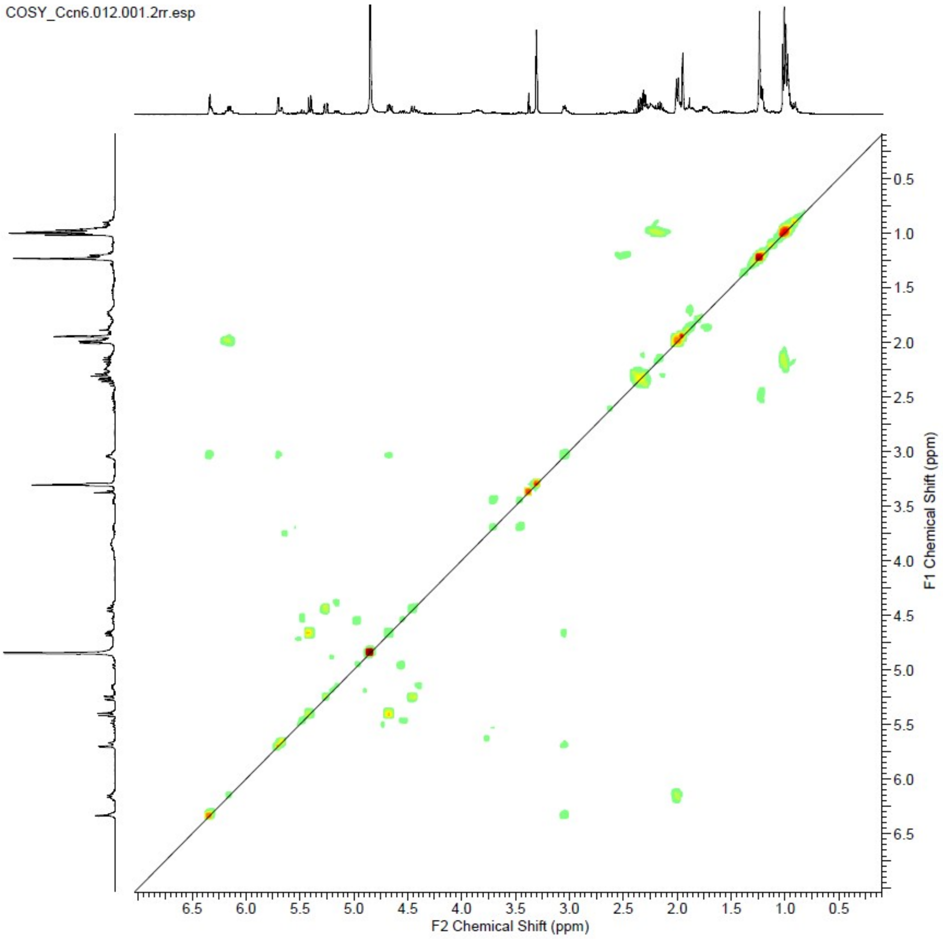

Figure S12: COSY spectrum of compound **4** in CD_3OD.

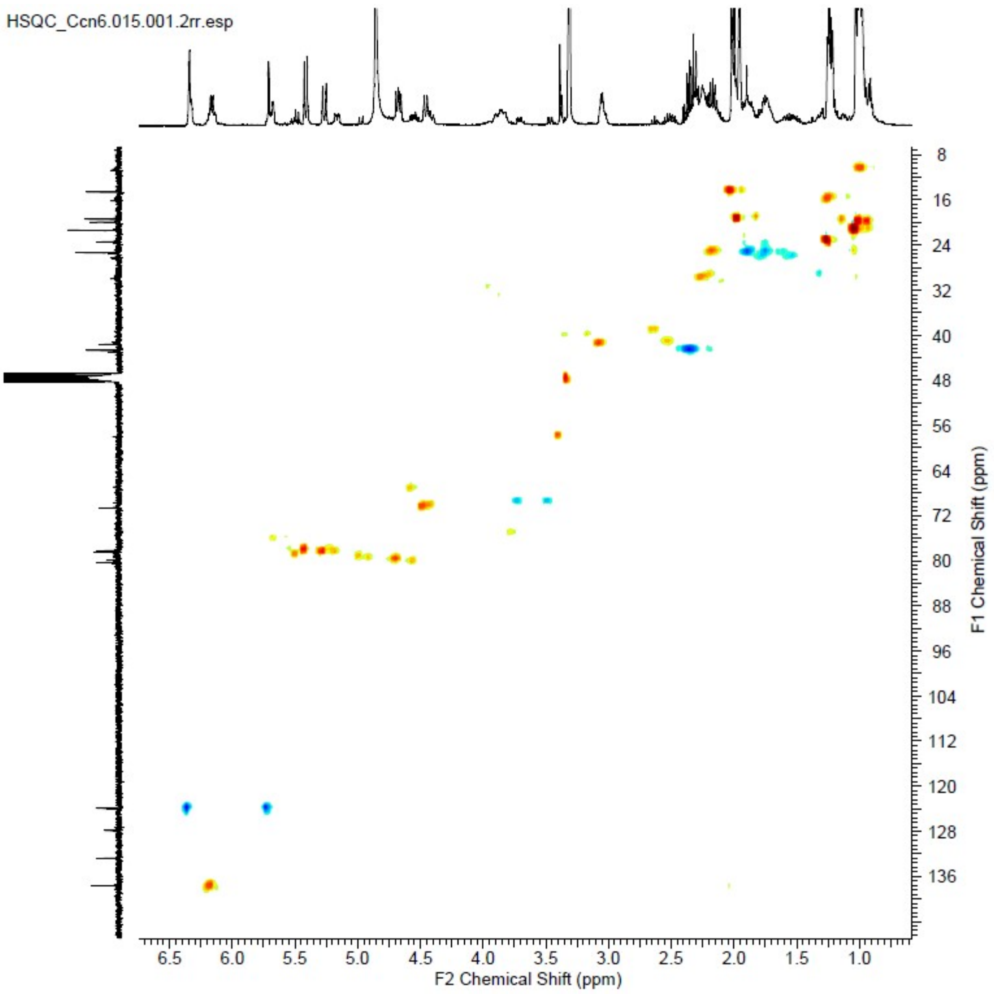

Figure S13: HSQC spectrum of compound **4** in CD_3OD.

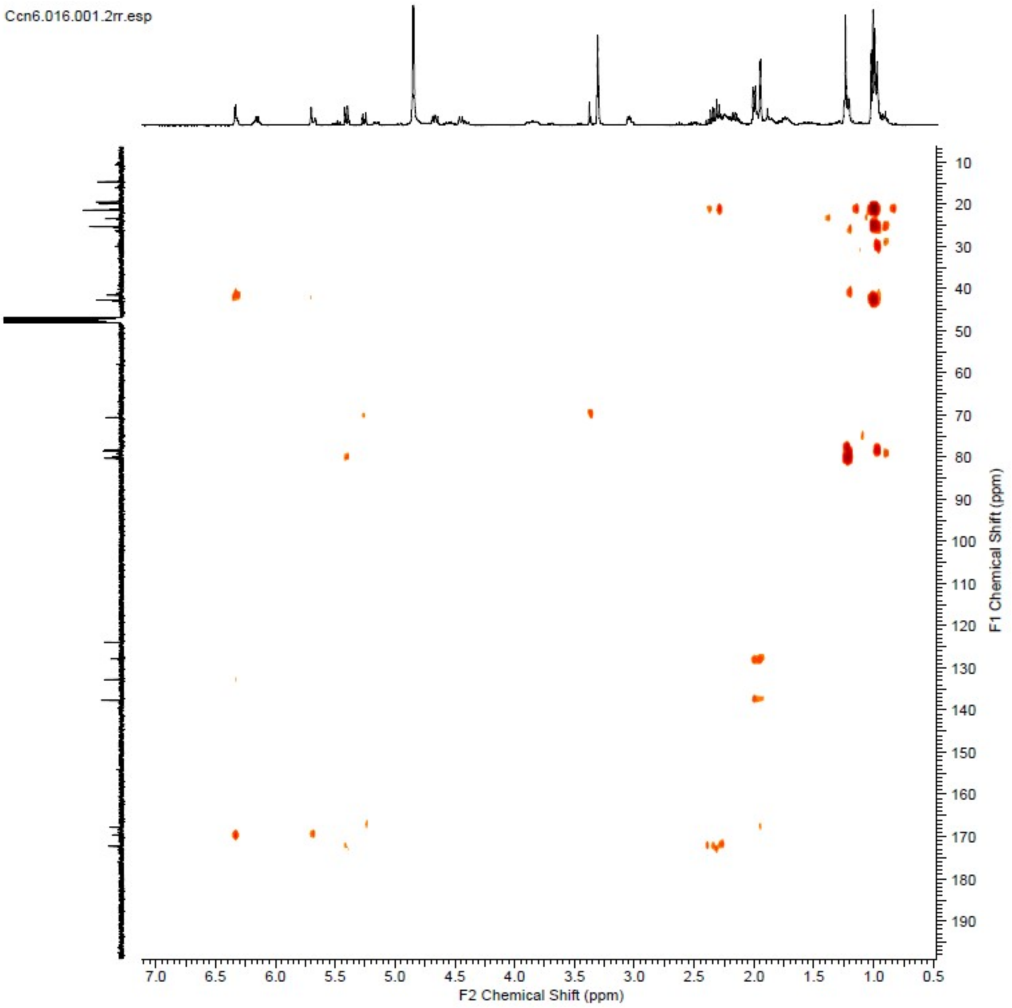

Figure S14: HMBC spectrum of compound **4** in CD_3OD.

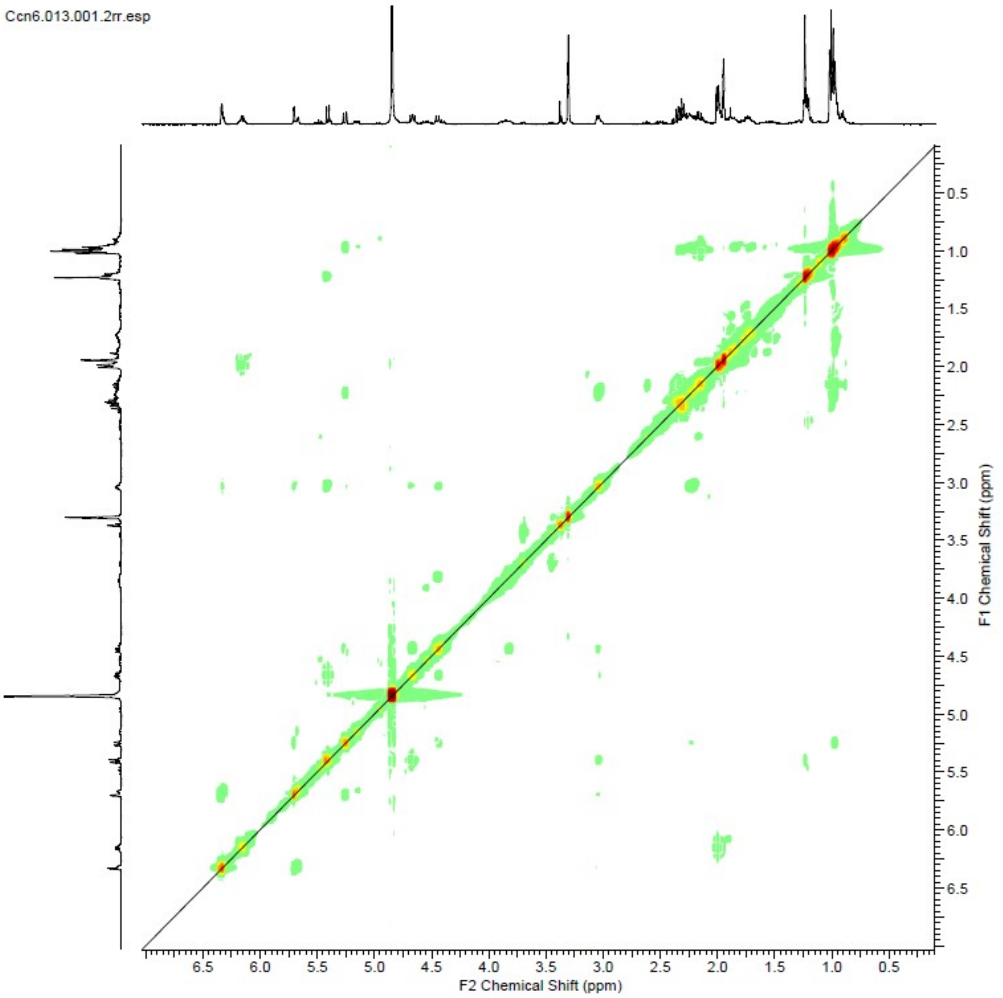

Figure S15: NOESY spectrum of compound **4** in CD_3OD.

Single Mass Analysis
Tolerance = 3.0 mDa / DBE: min = -1.5, max = 800.0
Element prediction: Off
Number of isotope peaks used for i-FIT = 3
Monoisotopic Mass, Even Electron Ions
10 formula(e) evaluated with 1 results within limits (up to 50 closest results for each mass)
Elements Used:
C: 0-150 H: 0-250 O: 2-4 Na: 1-1

Mass	Calc. Mass	mDa	PPM	DBE	Formula	i-FIT	i-FIT Norm	Fit Conf %	C	H	O	Na
273.1102	273.1103	-0.1	-0.4	5.5	C14 H18 O4 Na	1276.7	n/a	n/a	14	18	4	1

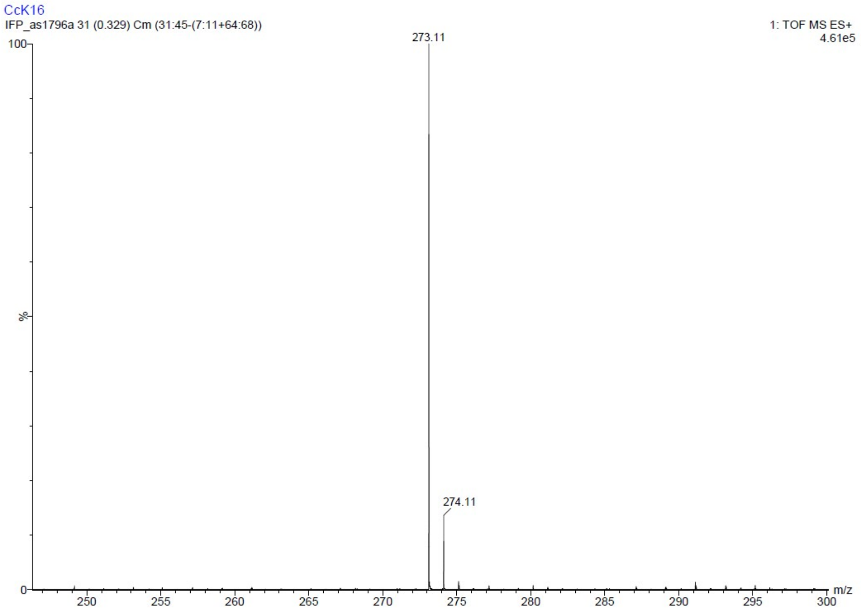

Figure S16: HRESIMS spectrum of compound **11**.

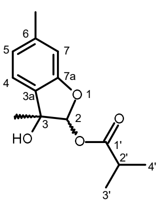

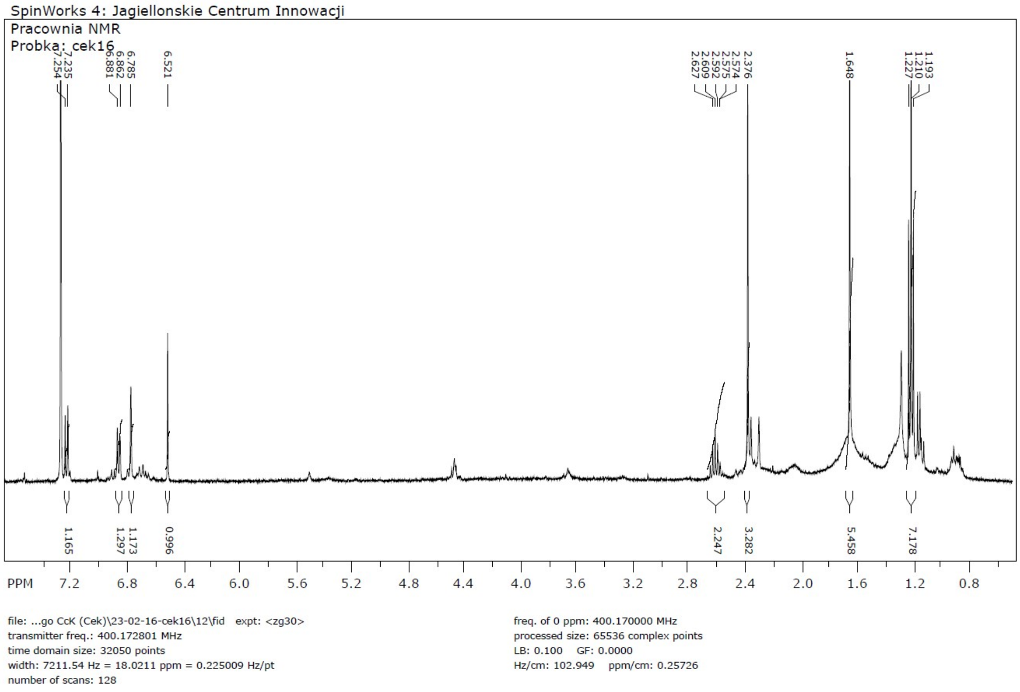

Figure S17: ^{1}H NMR spectrum of compound **11** in $CDCl_3$.

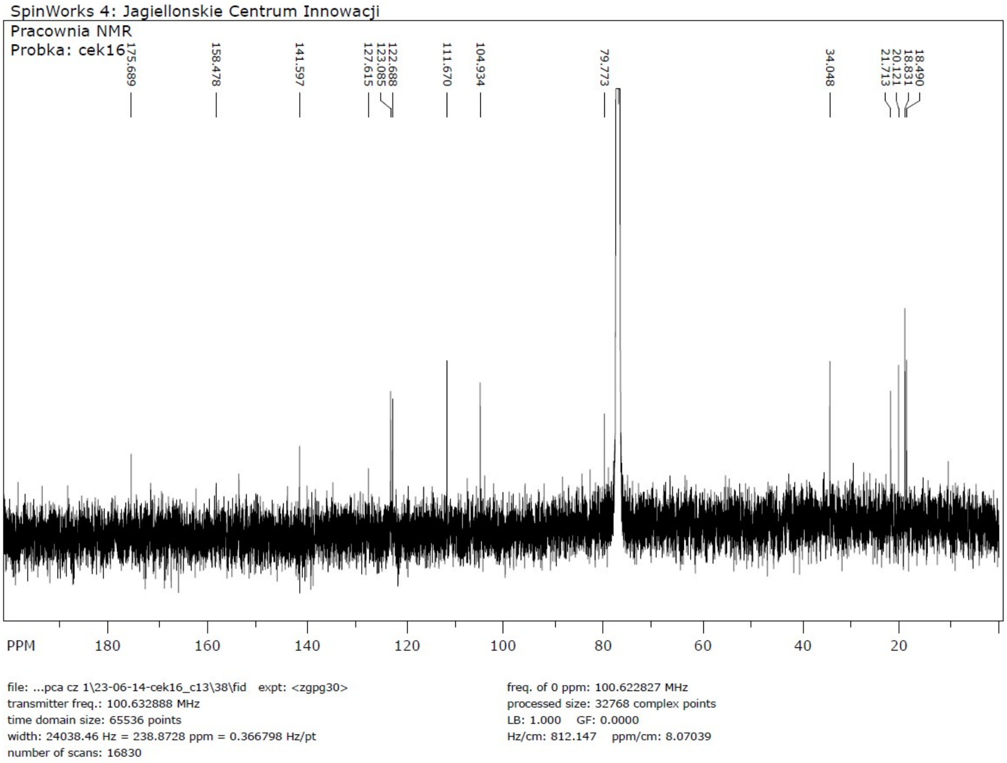

Figure S18: ^{13}C NMR spectrum of compound **11** in $CDCl_3$.

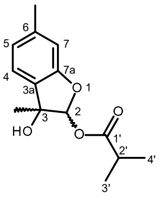

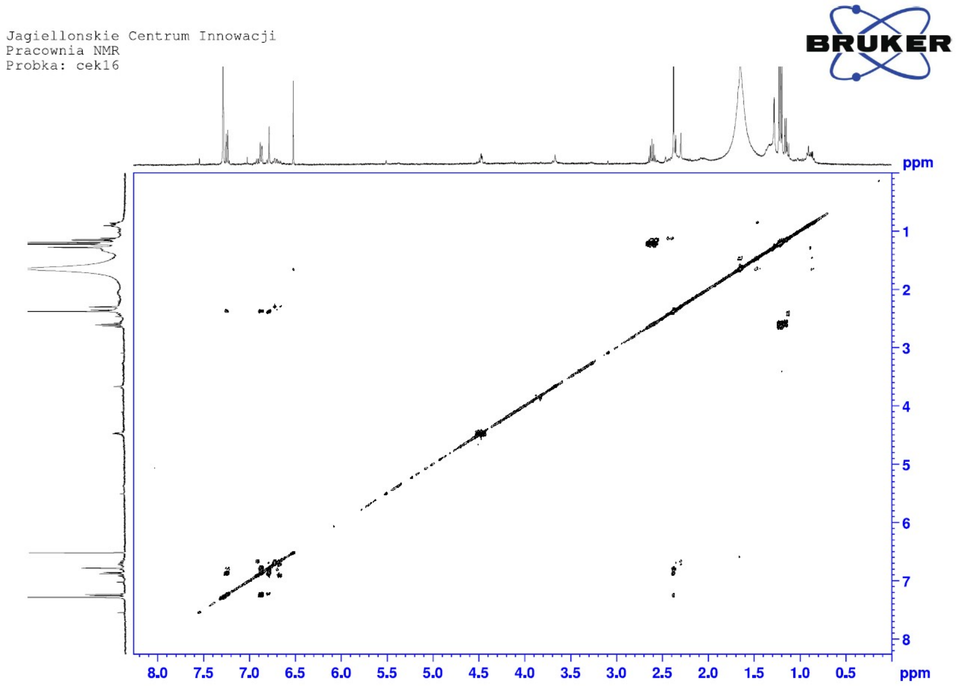

Figure S19: COSY spectrum of compound **11** in $CDCl_3$.

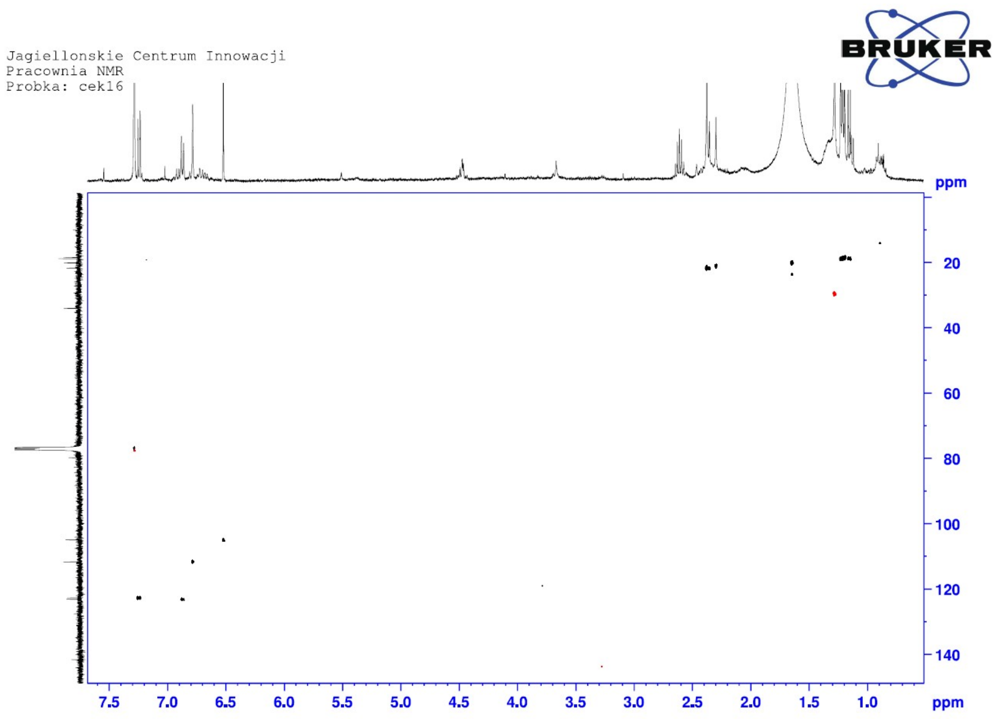

Figure S20: HSQC spectrum of compound **11** in $CDCl_3$.

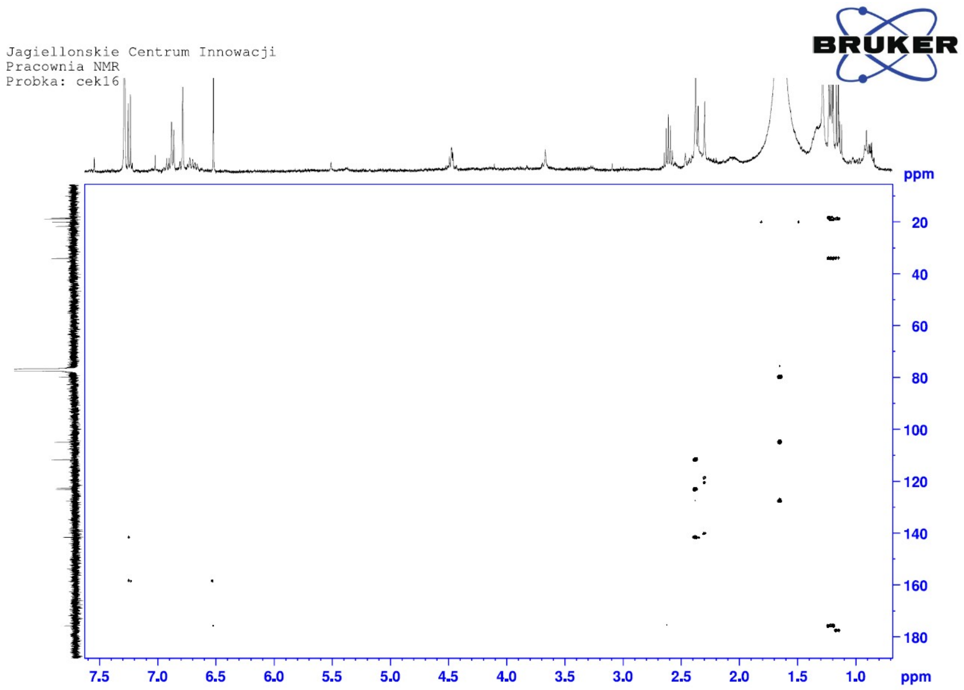

Figure S21: HMBC spectrum of compound **11** in $CDCl_3$.

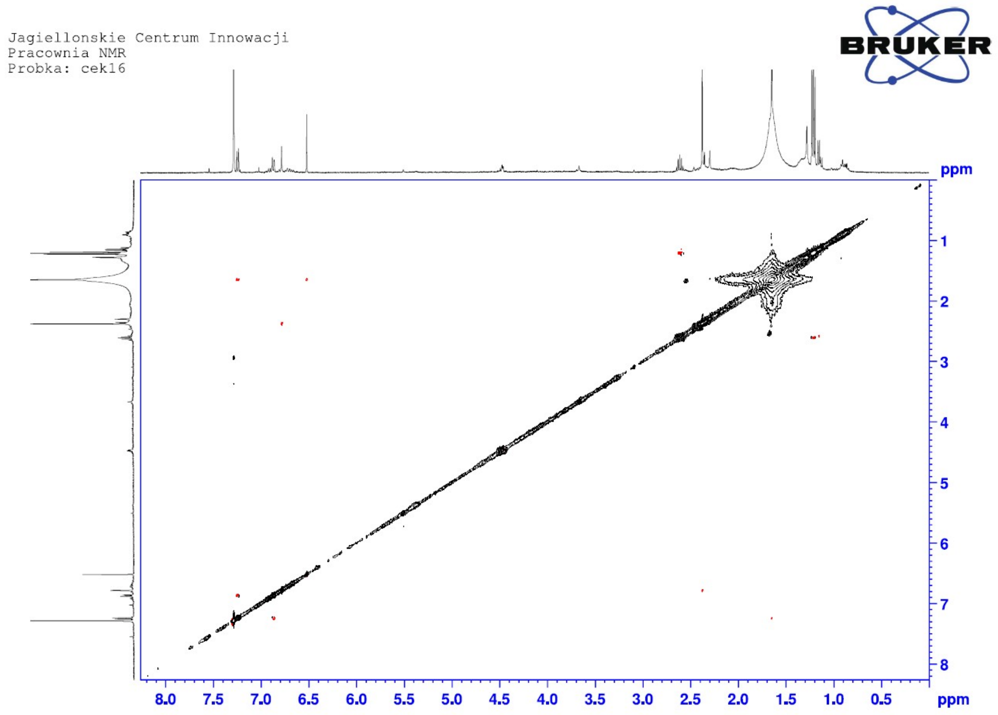

Figure S22: NOESY spectrum of compound **11** in $CDCl_3$.

Table S1. ^{13}C NMR (100.63 MHz) data of 8*α*-angeloyloxy-4*β*-hydroxy-5*β*-(3-methylbutyryloxy)-9-oxo-germacran-7*β*,12-olide (**1**), 8*α*-angeloyloxy-4*β*-hydroxy-5*β*-isobutyryloxy-9-oxo-germacran-7*β*,12-olide [30], 9*β*-angeloyloxy-4*β*,8*α*-dihydroxy-5*β*-(3-methylbutyryloxy)-3-oxo-germacran-6*α*,12-olide (**4**), and cardivarolide G [33] in CD_3OD.

**Position**	**Compound 1** δc **(ppm)**	8*α*-angeloyloxy-4*β*-hydroxy-5*β*-isobutyryloxy-9-oxo-germacran-7*β*,12-olide **[30]** δc **(ppm)**	**Compound 4** δc **(ppm)**	Cardivarolide G **[33]** δc **(ppm)**
1	21.4	22.9	25.4	25.4
2	36.2	37.7	33.0	33.2
3	33.1	34.5	217.7	217.8
4	72.4	73.8	80.4	80.4
5	77.3	78.6	78.2	78.1
6	72.0	73.3	79.9	80.0
7	44.9	46.3	41.7	41.6
8	78.4	79.8	70.6	70.3
9	212.1	213.5	78.5	78.7
10	41.3	42.7	30.0	29.9
11	133.5	134.0	132.8	132.8
12	169.4	170.7	169.6	169.7
13	126.0	127.4	123.9	123.9
14	19.5	20.9	20.0	20.0
15	23.5	24.8	23.5	23.4
1'	173.4	178.7	172.4	167.1
2'	42.2	34.9	42.8	127.5
3'	25.1	19.2	25.4	138.4
4'	21.4	19.2	21.4	19.3
5'	21.4		21.4	14.6
1"	165.7	167.1	167.8	173.2
2"	126.0	127.4	127.9	43.2
3"	141.7	143.1	137.7	25.4
4"	19.2	20.7	19.5	21.4
5"	14.7	16.1	14.7	21.5

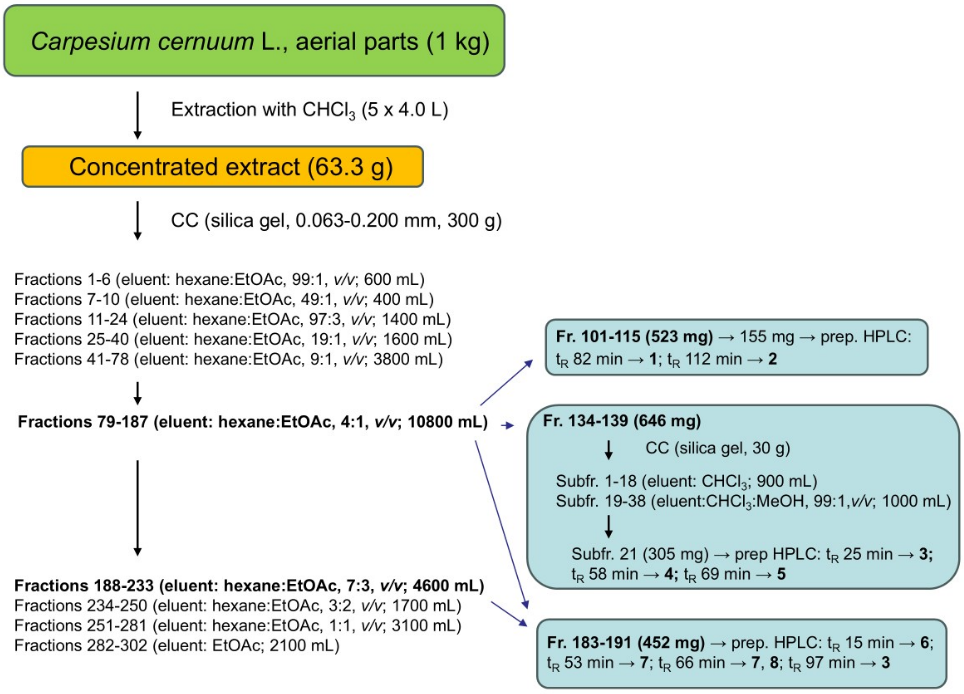

Figure S23: Fractionation scheme: A flowchart representing the chromatographic separation of a crude chloroform extract from aerial parts of *Carpesium cernuum* L. (EtOAc: ethyl acetate; MeOH: methanol; conditions for the preparative HPLC separations are specified in section 4.6.1. of the main text).

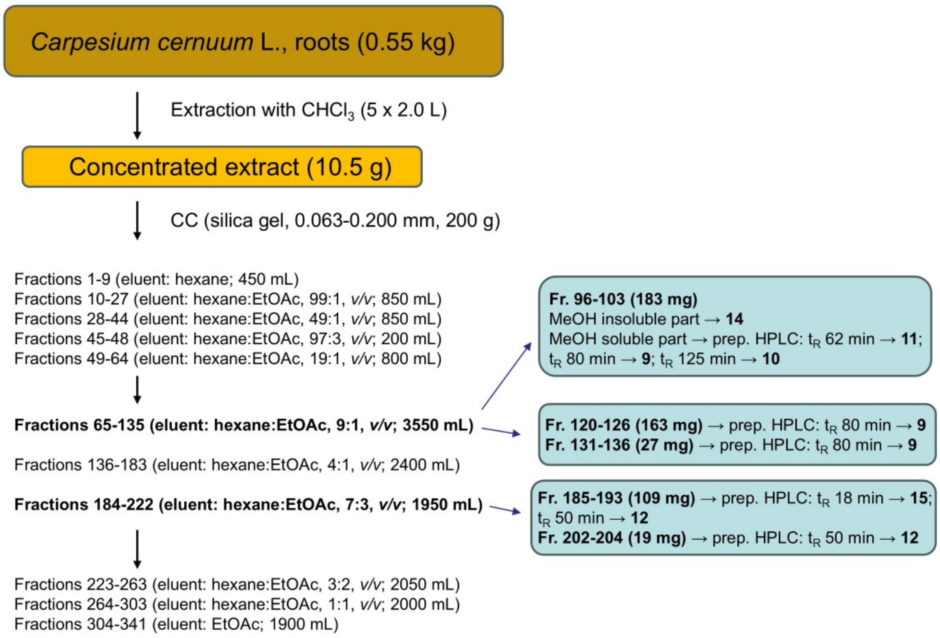

Figure S24: Fractionation scheme: A flowchart representing the chromatographic separation of a crude chloroform extract from roots of *Carpesium cernuum* L. (EtOAc: ethyl acetate; MeOH: methanol; conditions for the preparative HPLC separations are specified in section 4.6.2. of the main text).
